# Genome- and transcriptome-wide identification of trehalose-6-phosphate phosphatases (*TPP*) gene family and their expression patterns under abiotic stress and exogenous trehalose in soybean

**DOI:** 10.1186/s12870-023-04652-7

**Published:** 2023-12-12

**Authors:** Wenjing Shao, Xinlin Zhang, Zhiheng Zhou, Yue Ma, Duo Chu, Lei Wang, Yiming Yang, Lin Du, Yanli Du, Jidao Du, Qiang Zhao

**Affiliations:** 1 College of Agriculture, Heilongjiang Bayi Agricultural University, Daqing, 163319 Heilongjiang China; 2 National Coarse Cereals Engineering Research Center, Daqing, Heilongjiang China

**Keywords:** Trehalose-6-phosphate phosphatase (TPP), Soybean, Expression pattern, Abiotic stress, Saline-alkali stress, Trehalose

## Abstract

**Background:**

Trehalose-6-phosphate phosphatase (TPP) is an essential enzyme catalyzing trehalose synthesis, an important regulatory factor for plant development and stress response in higher plants. However, the TPP gene family in soybean has not been reported.

**Results:**

A comprehensive analysis of the TPP gene family identified 18 *GmTPP*s classified into eight groups based on the phylogenetic relationships and the conservation of protein in six monocot and eudicot plants. The closely linked subfamilies had similar motifs and intron/exon numbers. Segmental duplication was the main driving force of soybean *GmTPP*s expansion. In addition, analysis of the cis-regulatory elements and promoter regions of *GmTPPs* revealed that *GmTPPs* regulated the response to several abiotic stresses. Moreover, RNA-seq and qRT-PCR analysis of the tissue-specific *GmTPPs* under different abiotic stresses revealed that most *GmTPPs* were associated with response to different stresses, including cold, drought, saline-alkali, and exogenous trehalose. Notably, exogenous trehalose treatment up-regulated the expression of most *TPP* genes under saline-alkali conditions while increasing the carbohydrate and trehalose levels and reducing reactive oxygen species (ROS) accumulation in soybean sprouts, especially in the saline-alkali tolerant genotype. Furthermore, the interaction network and miRNA target prediction revealed that *GmTPP*s interacted with abiotic stress response-related transcription factors.

**Conclusions:**

The findings in this study lay a foundation for further functional studies on *TPP*-based breeding to improve soybean development and stress tolerance.

**Supplementary Information:**

The online version contains supplementary material available at 10.1186/s12870-023-04652-7.

## Background

Trehalose, a non-reducing disaccharide composed of two α- glucose molecules, is widely found in bacteria, yeast, invertebrates, protozoa, and higher plants [1–3]. It serves as an energy source during intracellular metabolism and also acts as a signaling molecule that regulates pathways involved in energy metabolism [4]. In plants, stress tolerance is significantly improved by regulating sugar accumulation, modulating the activity of sugar transporters, and sugar metabolism [5]. Trehalose accumulation is observed in many plant species under various abiotic stress conditions such as high and low temperatures, drought, salt, and high osmotic pressure. This accumulation is believed to have important evolutionary roles in protecting cellular structuresand biologically active substances like nucleic acids, membranes, and proteins [6]. However, plant response to abiotic stress is complex, involving many physiological and biochemical processes and molecular mechanisms, such as the differential expression of stress-related genes in various pathways [7].

The synthesis of trehalose in plants follows a highly conserved pathway that involves two enzymes. First, trehalose 6-phosphate synthase (TPS) catalyzes the synthesis of trehalose 6-phosphate (T6P) from uridine diphosphate-glucose and glucose 6-phosphate. Subsequently, trehalose-6-phosphate phosphatase (TPP) dephosphorylates T6P to produce trehalose [8, 9]. In addition, TPP acts as a signaling molecule that regulates important metabolic and developmental processes in plants. For example, TPP inhibits the activity of sucrose nonfermenting-related kinase1, crucial in the transcriptional regulatory network under stress conditions and energy metabolism [10].

Members of the TPP genes family encode TPP enzymes, and with the advant of genome sequencing technology, an increasing number of *TPP* gene family members have been identified in various plants. For example, 10 *TPP* gene family members (*AtTPPA-G*) have been identified in *Arabidopsis* [11], 13 (*OsTPP1-13*) in rice (*Oryza sativa*) [12], 79 (*GaTPP1-17*, *GrTPP1-12*, *GbTPP1-26*, *Gh1-24*) in cotton (*Gossypium spp*) [13], and 11 (*ZmTPPA1.1–1.2, Zm TPPB1.1–1.6, ZmTPPB2.1–2.2*) in maize (*Zea mays*) [14]. The TPP structure is highly conserved in plants, with specific structural domains containing only phosphatase domains. Furthermore, while TPP gene family members exhibit similar enzymatic activities, they display different patterns of differential expression, suggesting their involvement in specific tissues, stages, or processes [15].

Several *TPP* genes have been implicated in plant responses to abiotic stresses. Specifically, the overexpression or mutation of certain *TPP* genes has been shown to significantly impact plant growth, development, and tolerance to abiotic stress. For example, a mutation in the maize *RAMOSA3 TPP* gene has been linked to extensive inflorescence branching [16]. Similarly, the overexpression of *AtTPPI* or *AtTPPF* has been found to enhanced drought tolerance in *Arabidopsis* [17, 18]. In addition, the overexpression of *AtTPPD*, the only *TPP* family gene related to salt stress in *Arabidopsis*, which regulates sugar metabolism under salt stress, led to a significant accumulation of starch and soluble sugar [15]. In rice, the overexpression of *OsTPP1* or *OsTPP3* has been found to enhance tolerance to both salt and drought stresses [19, 20]. Additionally, the overexpression of *Mads6*, a promoter driving *OsTPP1* expression in maize, has been shown to significantly improves maize yield under drought conditions [21].

Soybean (*Glycine max*) is an economically important crop due to its high protein content and edible oil. However, soybean is susceptible to various abiotic stresses, which can negatively impacttheir growth and reduce crop productivity [22, 23]. Currently,, there is no research on *TPP* genes in soybean. In this study, we aimed to identify, describ, and phylogenetically classified 18 putative *TPP* genes in soybean. In addition, we also analyzed their gene structure, motifs, conserved structures, replication patterns, interaction networks, tissue-specific expression patterns, and *cis*-elements. Furthermore, we investigated the expression patterns of *GmTPPs* under abiotic stresses using RNA-seq and Real-time quantitative polymerase chain reaction (qRT-PCR). Additionally,, we analyzed the tissue-specific expression of *GmTPPs* and examined the effects of exogenous trehalose treatment on saline-alkali tolerance of soybean sprouts. This comprehensive analysis of the *GmTPP* gene family will serve as a foundation for further studies on their functions in soybean.

## Results

### Genome-wide identification and fundamental analysis of GmTPP genes

BLASTp search of the *GmTPP* gene members using 10 *Arabidopsis* protein sequences as the query identified 18 putative *TPP* genes in the soybean genome (Additional file 2: Table S1). The 18 *TPP* genes in the reference soybean genome (*Glycine max Wm82.a2. v1*) were confirmed by the combined Pfam (Trehalose_PPase; Pfam: PF02358), CDD-search, and HMMER analysis. Subsequently, they were labeled *GmTPP1* – *GmTPP18* according to their orders on the chromosomes. The physical and chemical properties of these genes are indicated in Table 1. The GmTPP protein lengths ranged from 214 to 392 aa with molecular weights of 24.24 (GmTPP11) to 43.43 (GmTPP7) kDa. In addition, their theoretical isoelectric point ranged from 5.51 (GmTPP16) to 9.78 (GmTPP8). GRAVY was predicted to be -0.518 (GmTPP17) to -0.027 (GmTPP11), implying that these proteins were hydrophilic. Finally, subcellular localization prediction revealed that the *GmTPPs* were localized in the vacuole and chloroplast.

**Table 1:** Physicochemical properties of identified *GmTPPs* in soybean

Gene ID	Gene Name	Chr	Number of amino acids	Molecular weight	PI	GRAVY	Formula	Subcellular
			(aa)	(kDa)				Location
Glyma.04G103600.1	GmTPP1	chr4	371	41.44	9.35	-0.277	C_1852_H_2957_N_511_O_542_S_12_	Chloroplast
Glyma.04G119700.1	GmTPP2	ch4	382	42.05	5.29	-0.208	C_1863_H_2936_N_502_O_573_S_16_	Cytosol
Glyma.04G237900.1	GmTPP3	chr4	367	41.77	8.86	-0.297	C_1872_H_2956_N_504_O_548_S_15_	Chloroplast
Glyma.05G179800.1	GmTPP4	chr5	362	41.20	9.27	-0.448	C_1826_H_2911_N_513_O_536_S_18_	Chloroplast
Glyma.06G104800.1	GmTPP5	chr6	380	42.41	9.46	-0.221	C_1899_H_3036_N_522_O_553_S_12_	Chloroplast
Glyma.06G126300.1	GmTPP6	chr6	367	42.05	9.09	-0.327	C_1884_H_2974_N_512_O_550_S_14_	Chloroplast
Glyma.06G318400.1	GmTPP7	chr6	392	43.43	5.82	-0.258	C_1929_H_3039_N_523_O_581_S_18_	Cytosol
Glyma.07G165100.1	GmTPP8	chr7	285	32.37	9.78	-0.146	C_1468_H_2349_N_399_O_402_S_11_	Chloroplast
Glyma.08G137500.1	GmTPP9	chr9	367	41.47	9.21	-0.476	C_1837_H_2931_N_519_O_538_S_18_	Chloroplast
Glyma.09G231400.1	GmTPP10	chr10	389	43.42	8.54	-0.476	C1_837_H_2931_N_519_O_538_S_18_	Cytosol
Glyma.11G168300.1	GmTPP11	chr11	214	24.24	9.61	-0.027	C_1940_H_3058_N_524_O_576_S_15_	Chloroplast
Glyma.11G239300.1	GmTPP12	chr11	363	41.04	9.31	-0.45	C_1103_H_1781_N_291_O_304_S_8_	Chloroplast
Glyma.12G005200.1	GmTPP13	chr12	389	43.28	7.66	-0.279	C_1822_H_2901_N_511_O_537_S_15_	Chloroplast
Glyma.13G088300.1	GmTPP14	chr13	372	41.82	9.29	-0.353	C_1933_H_3041_N_521_O_576_S_15_	Chloroplast
Glyma.14G171700.1	GmTPP15	chr14	379	42.54	9.47	-0.445	C_1869_H_2988_N_508_O_550_S_14_	Chloroplast
Glyma.16G025600.1	GmTPP16	chr16	313	35.20	5.51	-0.267	C_1888_H_3020_N_528_O_559_S_15_	Cytosol
Glyma.17G138700.1	GmTPP17	chr17	362	41.04	5.79	-0.518	C_1566_H_2471_N_413_O_475_S_16_	Cytosol
Glyma.18G018100.1	GmTPP18	chr18	364	41.07	9.31	-0.441	C_1808_H_2843_N_501_O_560_S_15_	Chloroplast

### Chromosome distribution and phylogenetic analysis of GmTPP genes in soybean

**Fig. 1:**
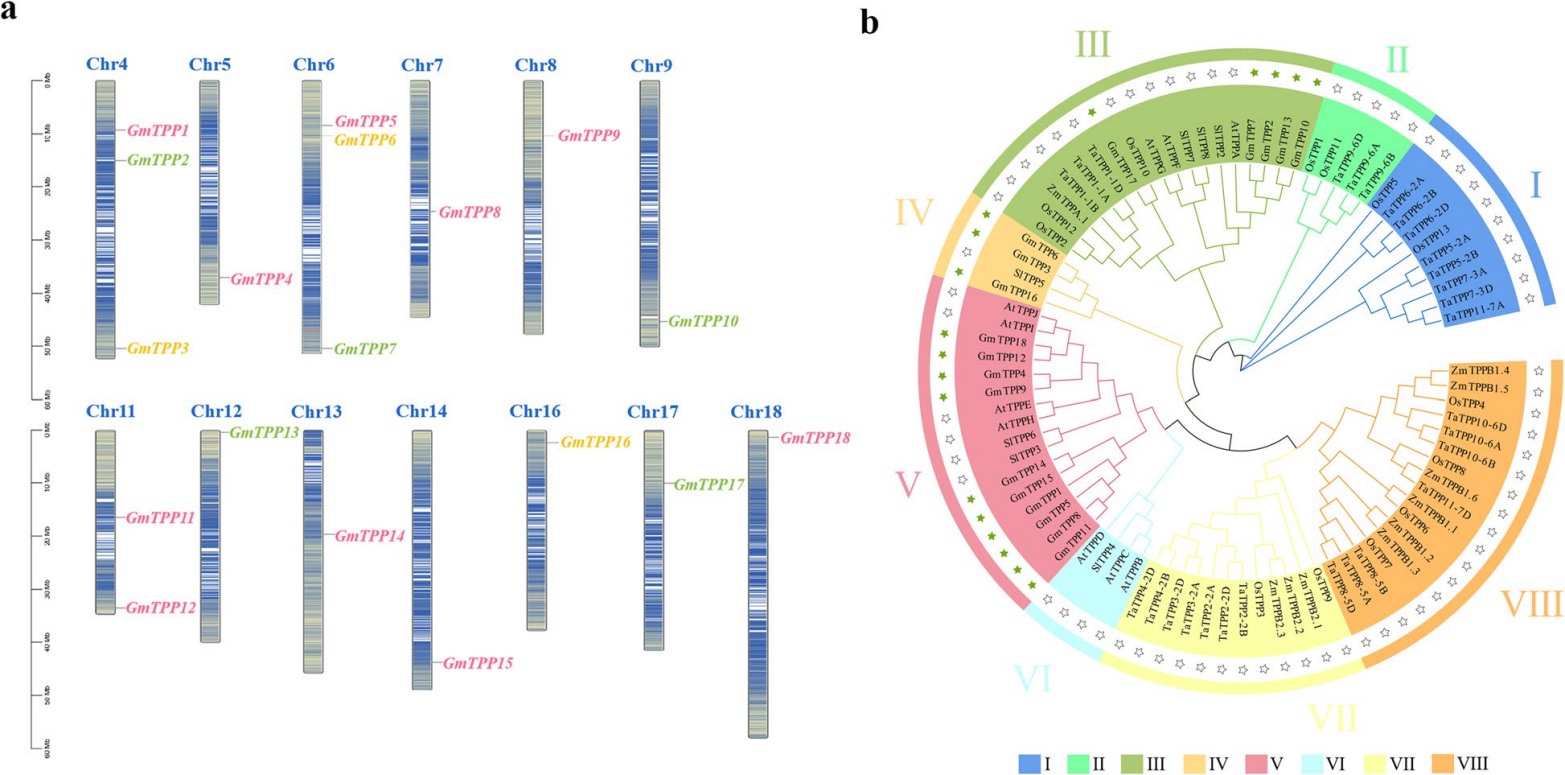
Chromosomal distribution and phylogenetic tree of *GmTPP*s. **a** Chromosomal distribution of *GmTPP*s. **b** Phylogenetic tree of *GmTPP*s from soybean, *Arabidopsis,* rice, wheat, tomato and maize. Different colors represent different groups. The blue lines indicate gene densit, filled stars represent GmTPPs genes and empty stars represent TPPs genes of other speciese

The chromosome maps based on the genomic sequences revealed the genomic distribution of *GmTPPs* on soybean chromosomes (Fig. 1a). The maps revealed that the TPP family members were evenly distributed on 13 chromosomes in the soybean genome.

Phylogenetic analysis based on the 87 TPP protein sequences from six plant species, including monocotyledonous angiosperms, wheat (*Triticum aestivum*), rice and maize, and the dicotyledonous angiosperms, *Arabidopsis*, tomato and soybean categorized the TPPs into eight subfamilies (I-VIII) (Fig. 1b; (Additional file 3: Table S2)). Subfamily III was comprised of *TPP* genes from the six plant species, while subfamily V comprised only *TPP* genes from the dicotyledonous angiosperms. Subfamilies I, II, VII, and VIII comprised *TPP* genes from different monocotyledonous angiosperms species, while subfamily VI had three *TPP* genes from *Arabidopsis* and one from tomato*.* The phylogenetic analysis revealed five, three, and ten GmTPPs in the subfamilies III, IV, and V, respectively. Most of the soybean TPP proteins were closed related to those of *Arabidopsis* and tomato. This implies that the differentiation of *TPP* genes in dicotyledonous angiosperms occurred later than in monocotyledonous angiosperms*.*

### GmTPPs conserved motif, gene structure, and domain analysis

**Fig. 2:**
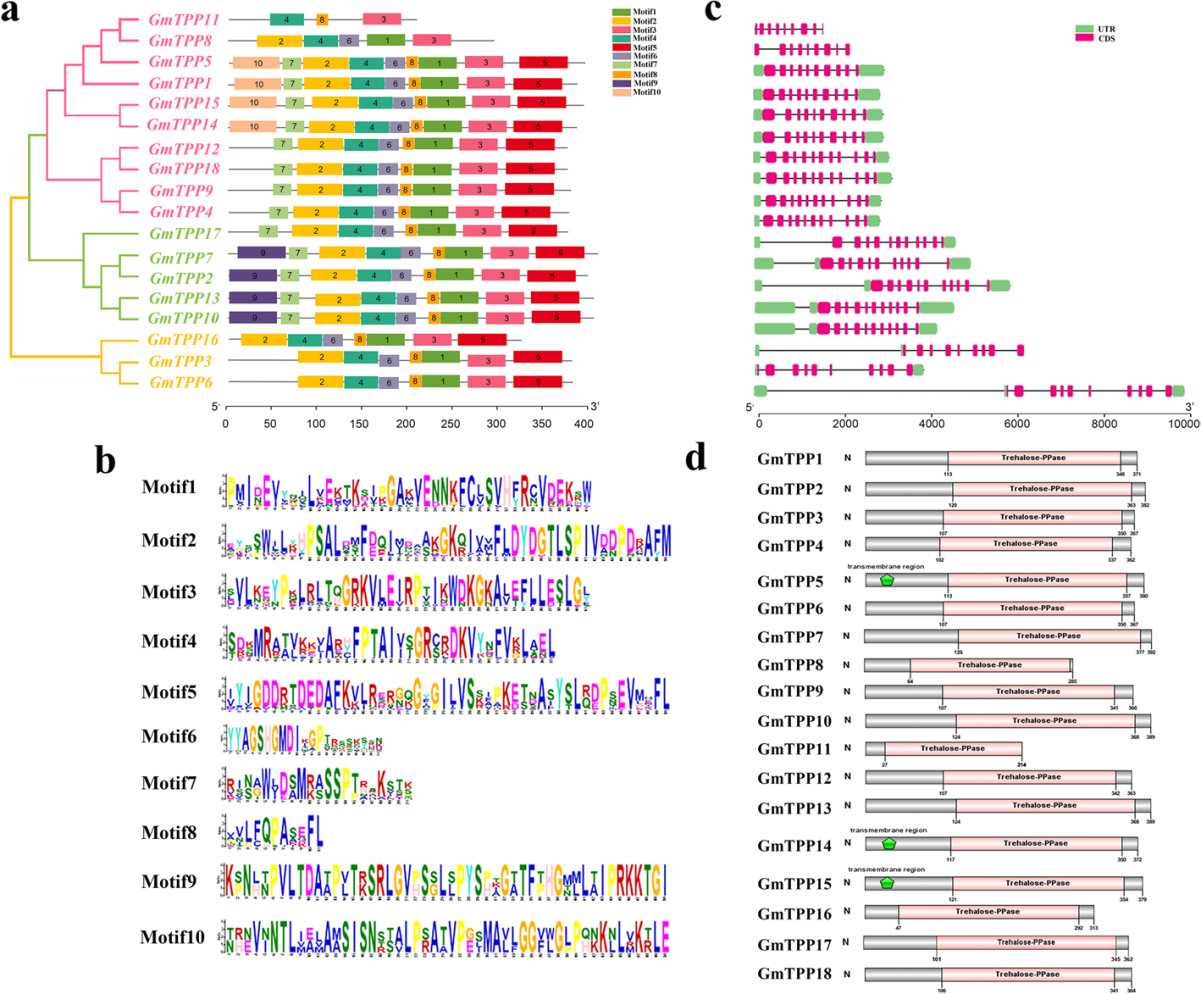
Phylogenetic tree, protein motifs, gene structures and protein conserved domain of GmTPPs. **a** Phylogenetic tree of GmTPPs in soybean and ten conserved motifs of GmTPPs proteins, each small box indicating a motif. **b** Conserved sequence of each motif. **c** The structures of intron and exon and untranslated regions (UTR) are shown in black line, green and pink boxes, respectively. **d** Conserved domain of GmTPPs protein in soybean

Domain analysis of the 18 full-length GmTPP protein sequences divided these proteins into three separate subfamilies (Fig. 2a). Motifs 3 and 4 were present in all the 18 GmTPPs proteins, implying that the two domains were highly conserved in *TPP* genes (Fig. 2a). Motifs 1, 2, 5, 6, and 8 existed simultaneously in sisteen GmTPP members, while Motifs 9 and 10 existed only in four GmTPP members. Overall, motifs 1–8 were present in most members. The conserved sequences of motifs 1–10 are shown in Fig. 2b.

Gene structure analysis revealed that *GmTPP* exons ranged from 9 to 11 and introns from 8 to 11, with genes clustered together having similar structures (Fig. 2c). *GmTPP* genes in the same subfamily had similar gene structures (intron number and exon length), particularly those of subfamily V, which had 11 introns in soybean. *GmTPP6* had the longest gene length, implying a different evolution pattern with variant characteristics. Furthermore, the Pfam analysis revealed that all the GmTPP proteins contained a specific Trehalose PPase domain (PF02358). In addition, GmTPP5, GmTPP14, and GmTPP15 proteins in subfamily II had a transmembrane region (Fig. 2d). The 18 proteins also had similar 3D structures (Additional file 1: Fig S1). The 3D structure of these proteins lays the foundation for their biological function.

### Gene duplication and homology analysis of TPP genes

**Fig. 3:**
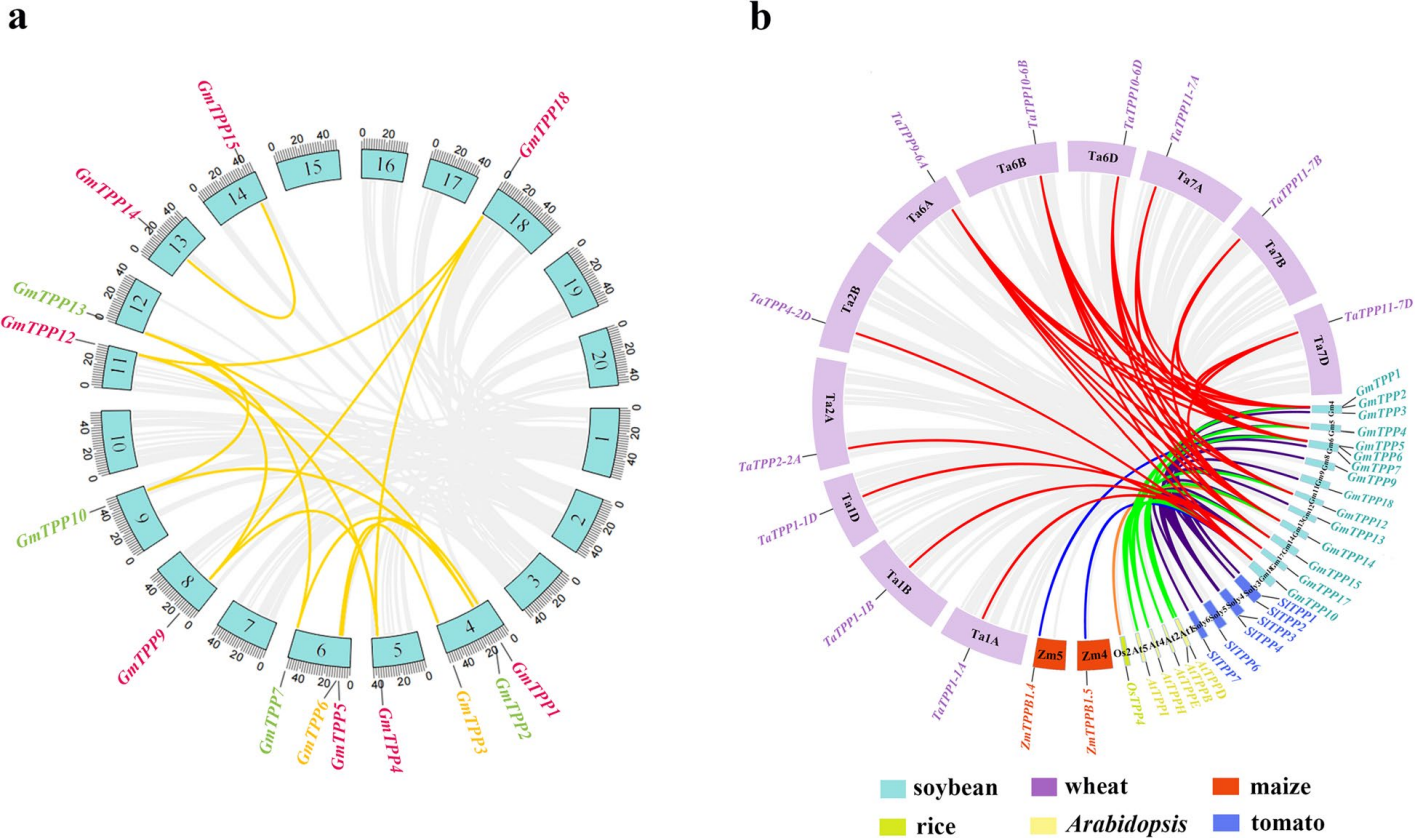
Collinear analysis of GmTPPs. **a** Collinearity analysis of TPP gene family in soybean. **b** Collinear analysis of GmTPPs with *Arabidopsis*, maize, rice, wheat, tomato. Different gene colors represent different groups

The evolutionary mechanism of the soybean TPP family was revealed based on the collinear relationships of the TPP gene family in dicots (soybean, *Arabidopsis*, tomato) and monocots (wheat, rice, and maize) (Fig. 3). Among the 18 *GmTPP* genes, 14 possible pairs of duplicated genes existed (Fig. 3a). Based on the gene repetition analysis, all the identified paralogous genes were segmental duplications (SD), implying that SD was the main expansion mechanism of the *GmTPP* gene family. The ratio of the non-synonymous mutation rate (Ka) to the synonymous mutation rate (Ks) was used to express the selection pressure analysis of coding sequences, with *Ka/Ks* < 1 indicating purified/negative selection and *Ka/Ks* > 1 Darwinian/positive selection. The *Ka/Ks* ratio ranged from 0.0109503 to 0.1057524, implying that purifying selection was important during gene replication (Additional file 4: Table S3). These findings suggest that fragment replication events had important effects on the diversity of *TPP* genes in soybean.

Furthermore, homology analysis revealed that seven *GmTPPs* and five *AtTPPs* were orthologous gene pairs, resulting in 13 syntenic relationships. *GmTPP5*, *GmTPP14*, and *GmTPP15* were collinear with *AtTPPD*, while *GmTPP10* was collinear with *AtTPPE*, implying that they may have similar functions.The *GmTPPs* were collinear with 26 relationships in tomato, while nine *GmTPP* genes were collinear with 33 relationships in wheat. In addition, two *GmTPPs*, two *ZmTPPs,* one *GmTPPs,* and one *OsTPPs* were orthologous gene pairs, resulting in syntenic relationships, respectively. The orthologous relationships between the soybean *TPP* genes and related genes from the six representative species, including three dicots (*Arabidopsis*, soybean, and tomato) and three monocots (rice, maize, and wheat) are illustrated in Fig. 3b.

### Promoter regions cis-acting regulatory elements analysis

**Fig. 4:**
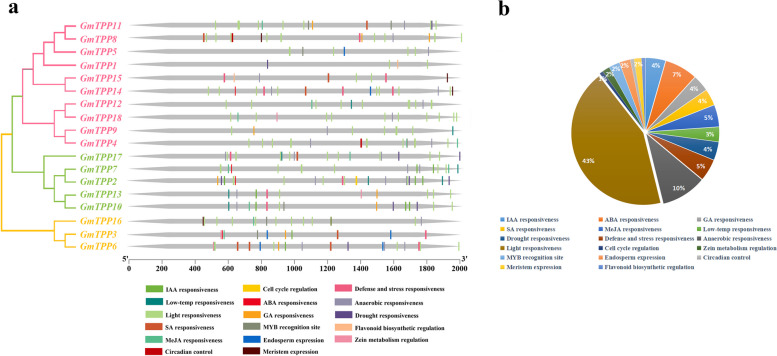
The Cis-regulatory analysis of GmTPPs. **a** Cis-regulatory elements in promoter region of *GmTPPs* genes and their proportions (**b**)

The CREs analysis identified 18 major CREs in the *GmTPPs* promoter sequence (Fig. 4a). The 18 major CREs were classified into four element categories, including phytohormones, cellular development, photoresponsive, and environmental stress (Additional file 5: Table S4)*.* Among them, the light-responsiveness CREs accounted for the largest proportion (44.4%), followed by the hormone-regulated elements (such as auxin, abscisic acid, gibberellins, salicylic acid, and methyl jasmonate), which accounted for 24.5%. The other two categories, including developmental and stress-related elements, accounted for 31.1% (Fig. 4b). The promoter regions of seven genes had low-temperature responsiveness elements, which contained all members of group III. In addition, there were nine genes involved in defense/stress, including four genes in group III (*GmTPP2, GmTPP10, GmTPP13,* and *GmTPP17*), two in group IV, and two in group V. Overall, genes in group III played an important role in defense/stress response. The promoter regions of six genes also had drought induction elements, of which three were in group III and the other three were in group V. The CREs analysis revealed that *GmTPPs* respond to several hormones and stresses, which may directly affect the stress response ability of *GmTPPs* under stress conditions. Among them, *GmTPP2* and *GmTPP15* were involved in response to several abiotic stress and hormones and the growth regulation of soybean.

### Expression pattern of GmTPP genes in different tissues

**Fig. 5:**
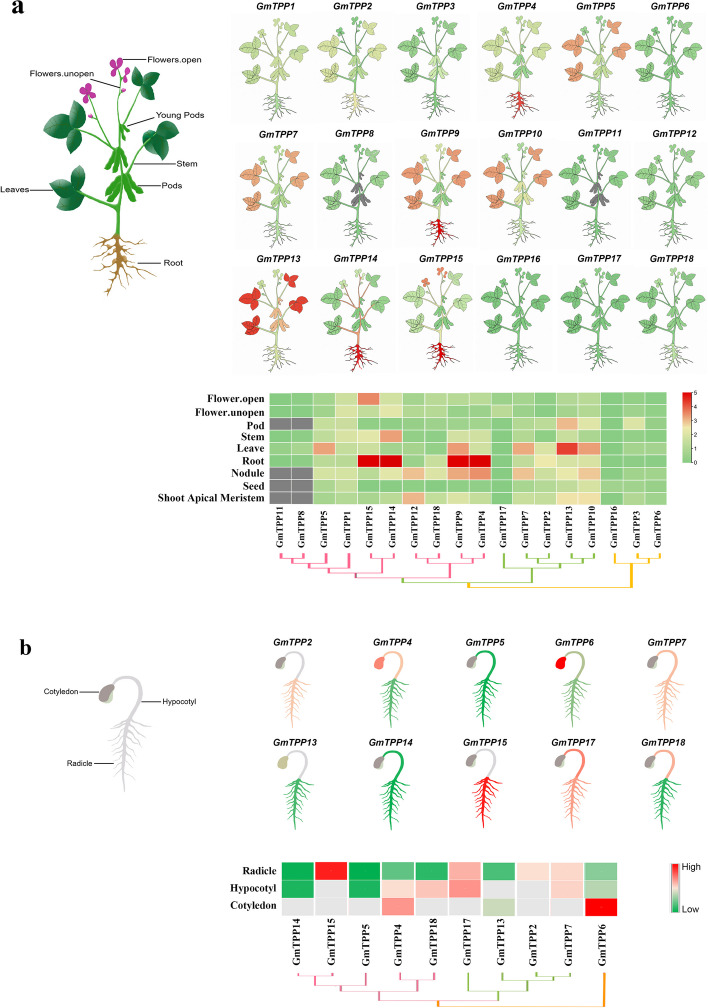
The expression levels of *GmTPPs* in different tissues. **a** The schematic diagram of different tissues of soybean and the expression of *GmTPPs* in different tissues. **b** The schematic diagram of different tissues of soybean sprouts and the expression level of *GmTPPs* in cotyledons, hypocotyls, and radicles. Red and green indicate high and low transcription levels, respectively. The expression levels of *GmTPPs* were analyzed using the 2^− △△Ct^ methods

The expression levels of *GmTPPs* in different tissues, including the flowers, stems, roots, nodules, shoots, leaves, pods, and seeds, were obtained from the Phytozome database. The expression levels of *GmTPPs* in the soybean tissues were significantly different (Fig. 5a). For example, *GmTPP4*, *GmTPP9*, *GmTPP14*, and *GmTPP15* were up-regulated in the roots, and *GmTPP13* in the leaves. In addition, the relative expression of *GmTPP5*, *GmTPP7*, *GmTPP9,* and *GmTPP10* was also significantly higher in the leaves than in the other organs or tissues. *GmTPP13* was also highly expressed in pods. In contrast, *GmTPP6*, *GmTPP12*, *GmTPP16*, *GmTPP17*, and *GmTPP18* were lowly expressed in all the plant tissues.

qRT-PCR analysis of the expression patterns of the 18 *GmTPPs* during the sprout stage revealed tissue-specific expression in soybean tissues (Fig. 5b). The expression pattern of 10 *GmTPPs* was detected in the soybean sprouts. *GmTPP2*, *GmTPP7*, *GmTPP15,* and *GmTPP17* in soybean radicles were highly expressed, with the highest expression level in *GmTPP15*. Besides, *GmTPP4* and *GmTPP6* expression levels in the soybean cotyledon were higher than in the radicle and hypocotyls. Similarly, *GmTPP7*, *GmTPP17,* and *GmTPP18* expression levels in the soybean hypocotyls were higher than in the radicle and cotyledon (Additional file 6: Fig S2). Taken together, *GmTPPs* expression in soybean is tissue-specific expressed during the sprout stage.

### Expression profiles of GmTPP genes under abiotic stresses

**Fig. 6:**
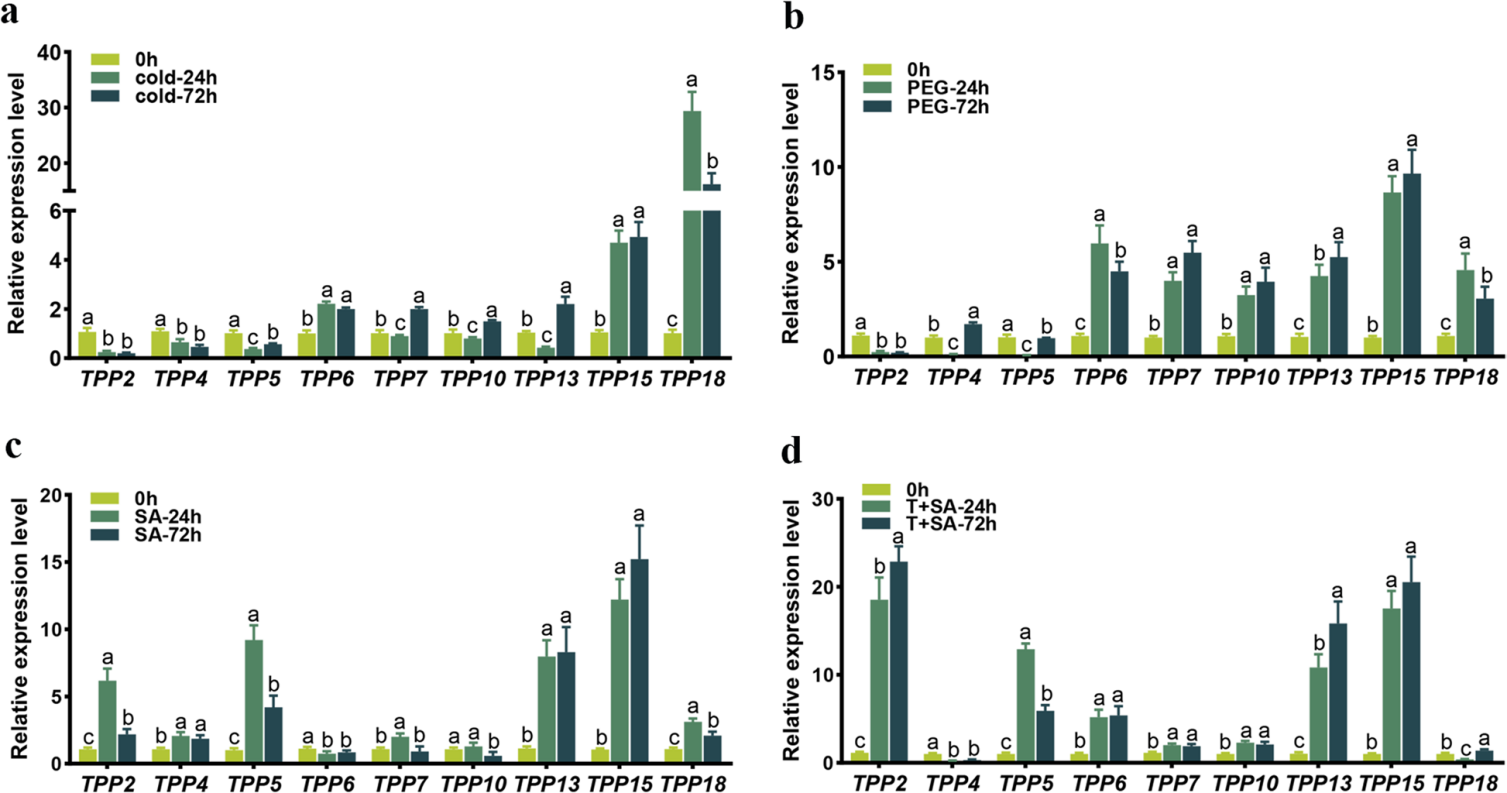
Expression profiles of soybean *GmTPPs* genes in response to various abiotic stress treatments. The various abiotic stress treatments including low temperature (**a**), drought (**b**), SA (**c**) and T + SA (**d**). Low temperature treatment at 4 ◦C for 24 h and 72 h; untreat indicates control plants; PEG: drought treatment; SA: saline-alkali treatment; T + SA: trehalose immersion plus saline-alkali treatment. The expression levels of *GmTPPs* were analyzed using the 2^−△△Ct^ methods

qRT-PCR analysis on the soybean sprouts treated with 4˚C, PEG, saline-alkali, and exogenous trehalose (T + SA) revealed the expression pattern of *GmTPPs* in response to abiotic stress (Fig. 6). Precisely, the expression levels of *TPP* family genes under the same treatment conditions were different. Besides, the expression patterns of the same genes under different treatment conditions differed.

The *GmTPP6*, *GmTPP7*, *GmTPP13, GmTPP15,* and *GmTPP18* expression were induced under 4˚C and drought (PEG) treatments, with peak expression at 72 h after treatment, implying that these genes are sensitive to cold and drought stresses (Fig. 6ab). Except for *GmTPP6* and *GmTPP7*, the expression levels of all the other genes increased to more than 6 folds under saline-alkali stress (Fig. 6c). The expression levels of *GmTPP13* and *GmTPP15* were increased with the exposure time to saline-alkali stress. *GmTPP2*, *GmTPP5*, *GmTPP6*, *GmTPP7*, *GmTPP13,* and *GmTPP15* were also up-regulated under exogenous trehalose treatment compared to under saline-alkali treatment (Fig. 6d). Moreover, *GmTPP4* and *GmTPP18* expression levels were decreased under exogenous trehalose treatment. At the same time, *GmTPP2* and *GmTPP5* responded only to saline-alkali and T + SA treatments, while *GmTPP6* and *GmTPP7* were downregulated only under saline-alkali stress. In addition, *GmTPP13* and *GmTPP15* were significantly up-regulated under the four abiotic stress conditions. In summary, the *GmTPP* genes were responsive to abiotic stress, with most genes responding (up-regulated) to saline-alkali stress. In addition, gene expression was significantly increased after trehalose treatment, implying that trehalose could alleviate the damage caused by saline-alkali stress.

### Validation of GmTPPs differential expression

**Fig. 7:**
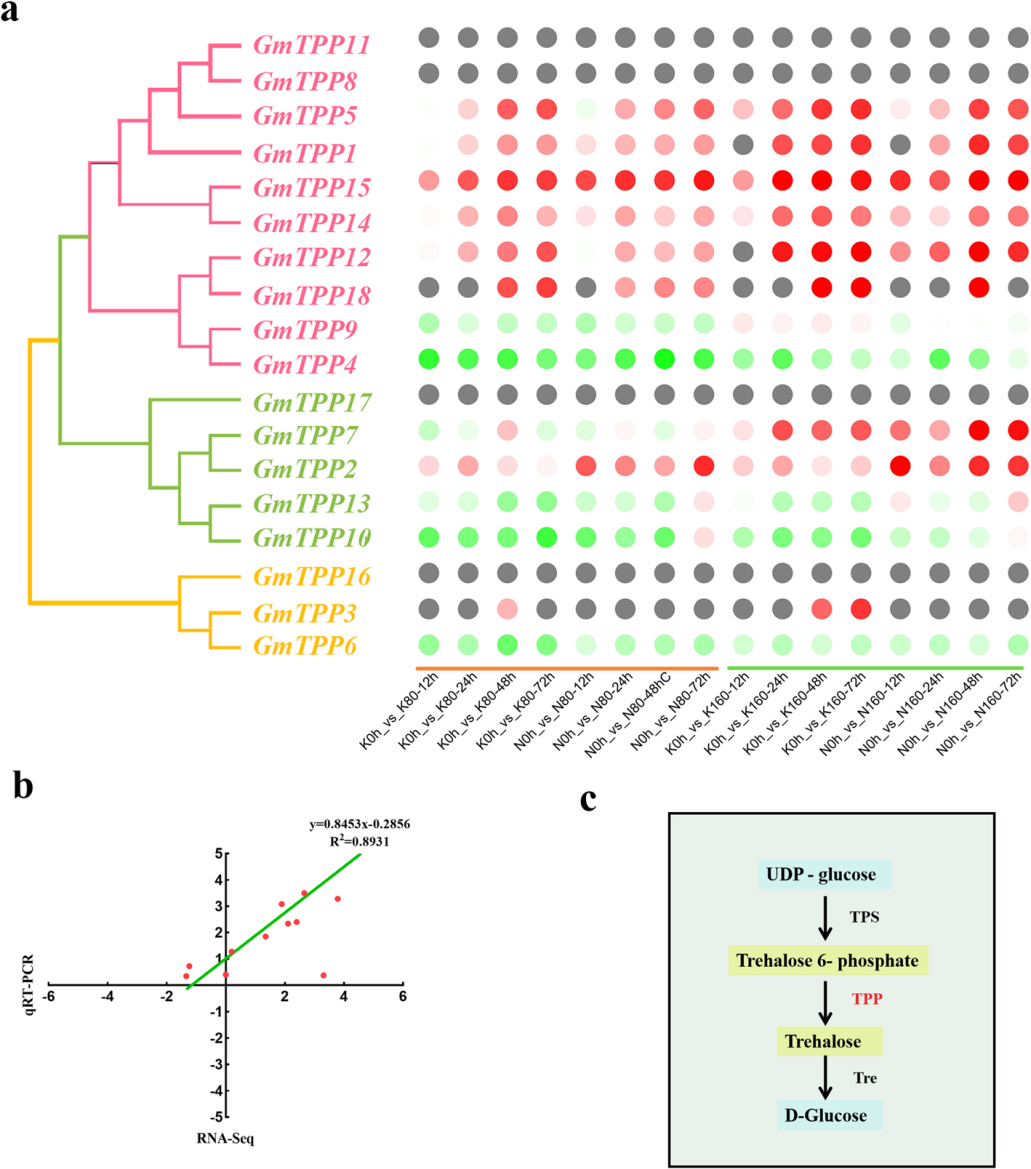
RNA-seq analysis of the two genotypess under saline-alkali stress. **a** Eighteen *GmTPPs* selected for qRT-PCR analysis and the accuracy of RNA-seq was verified. Red to green represent high to low expression. **b** Eighteen GmTPPs selected for qRT-PCR analysis and the accuracy of RNA-seq was verified. **c** GmTPPs enriched in trehalose biosynthetic pathway. The ‘K’ represent saline-alkali sensitive genotype and ‘N’ represent saline-alkali tolerant genotype

The integrated transcriptome analysis revealed the *GmTPPs* expression patterns in the radicle of the different soybean genotypes under saline-alkali stress (Fig. 7a). The raw data were submitted to the NCBI database. There was a significant difference in the expression of the two genotypes (N and K) GmTPPs genes under saline treatment. *GmTPP2, GmTPP5*, *GmTPP12*, *GmTPP14,* and *GmTPP15* were significantly up-regulated in the N genotype compared to K genotype. Among them, the expression levels of *GmTPP2*, *GmTPP7*, *GmTPP12,* and *GmTPP15* were more than threefold. In contrast, *GmTPP4*, *GmTPP6*, *GmTPP9*, *GmTPP10,* and *GmTPP13* were down-regulated under saline-alkaline stress. Validation of the RNA-seq results by qRT-PCR analysis of the expression levels of the 18 *GmTPP* genes revealed that their expression levels had the same trend based on RNA-seq and qRT-PCR analysis, with an R^2^ of 0.8931 (Fig. 7b). Furthermore, Go enrichment and KEGG pathway annotation analyses revealed that the starch and sucrose metabolism pathway was enriched. In particular, all the *GmTPPs* were enriched in the trehalose biosynthetic process (Fig. 7c).

### Exogenous trehalose in both genotypes and the expression of GmTPPs

**Fig. 8:**
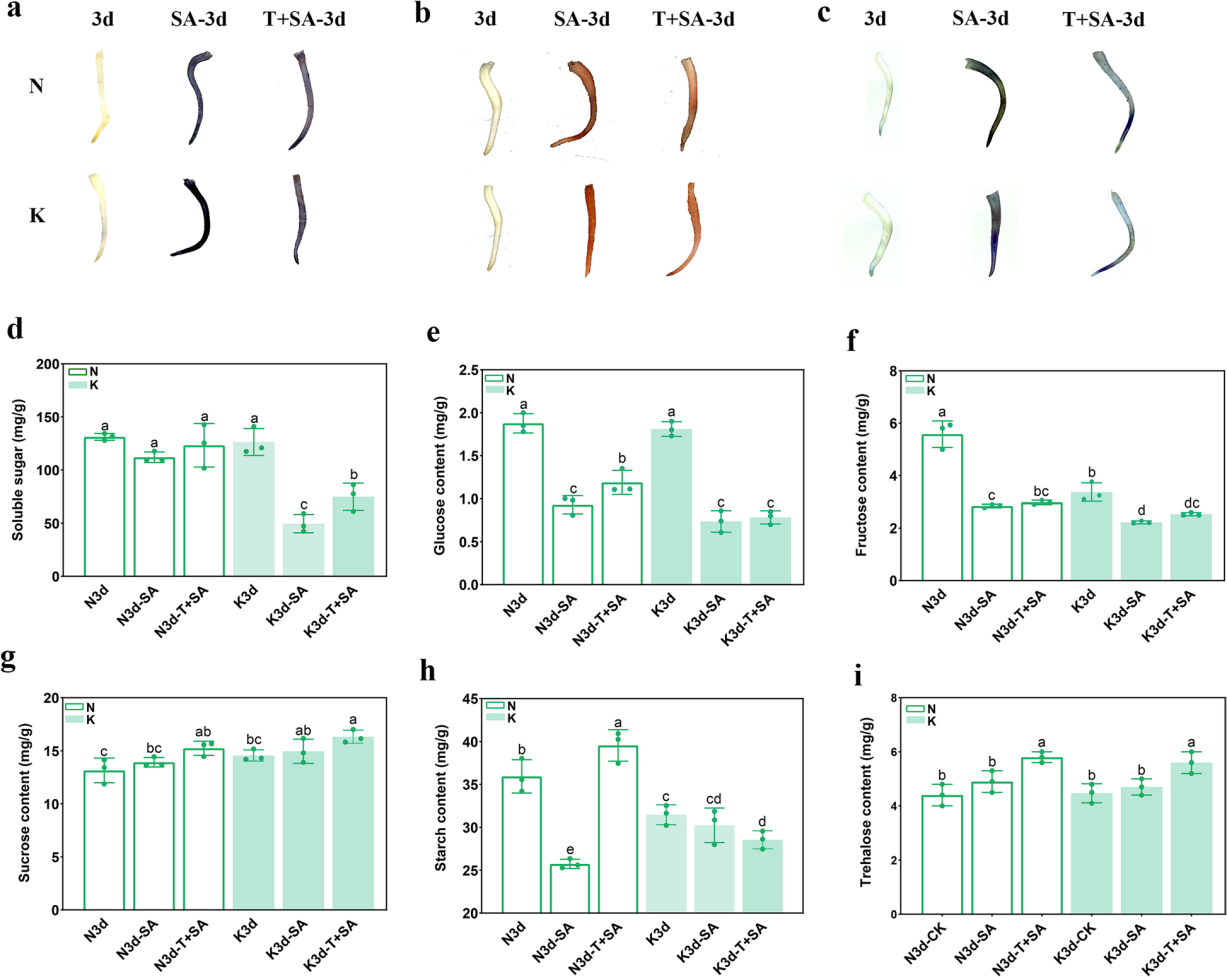
Effects of trehalose immersion on ROS and carbohydrate content of soybean sprouts under saline-alkali conditions. **a**-**c** was NBT, DAB and TB staining, respectively. **d** Soluble content. **e** Glucose content. **f** Fructose content. **g** Sucrose content. **h** starch conten. **i** Trehalose content. The results showed the mean ± SE of three replicates, and the different letters denote the significant difference among treatments (*p* < 0.05). 0 d, control; SA, 160 mM saline-alkali; T + , 10 mM trehalose + 10 mM saline-alkali

To investigate the effect of trehalose on soybean response to saline-alkali stress, the N and K soybean genotypes were treated with 10 μmol/L trehalose and 160 mmol/L mixed saline-alkali solution consisting of NaCl, Na_2_CO_3_, NaHCO_3_, and Na_2_SO_4_ (the molar ratio is 1:1:9:9) for 3 d, with the N and K soybean genotypes cultured in water serving as the control. The nitro blue tetrazolium (NBT), diaminobenzidine (DAB) and TB staining revealed that the active, hydrogen peroxide (H_2_O_2_) and superoxide anion (O_2_^−^) content in the ‘N’ genotype were greater than in the ‘K’ genotype under saline-alkali stress. In contrast, trehalose immersion resulted in a lower ROS accumulation compared to the control and saline-alkali treatment (Fig. 8d-i).

The trehalose levels were increased under saline-alkali stress, significantly increasing after trehalose immersion. However, the trends of soluble sugar, glucose, fructose, sucrose, and starch were inconsistent with trehalose levels (Fig. 8a-e). Under saline-alkali treatment alone, the soluble sugar, fructose, glucose, sucrose, and starch contents were decreased but increased under the T + SA treatment, especially in the saline-alkali tolerant genotype (N). Besides, the trehalose content under T + SA treatment was higher than under saline-alkali treatment (Fig. 8f). These results indicate that the energy metabolism of soybean was severely inhibited under saline-alkali stress but was alleviated by soaking in trehalose.

### Interaction network analysis of GmTPPs and transcription factors and the prediction of miRNAs encoded by TPP family genes

**Fig. 9:**
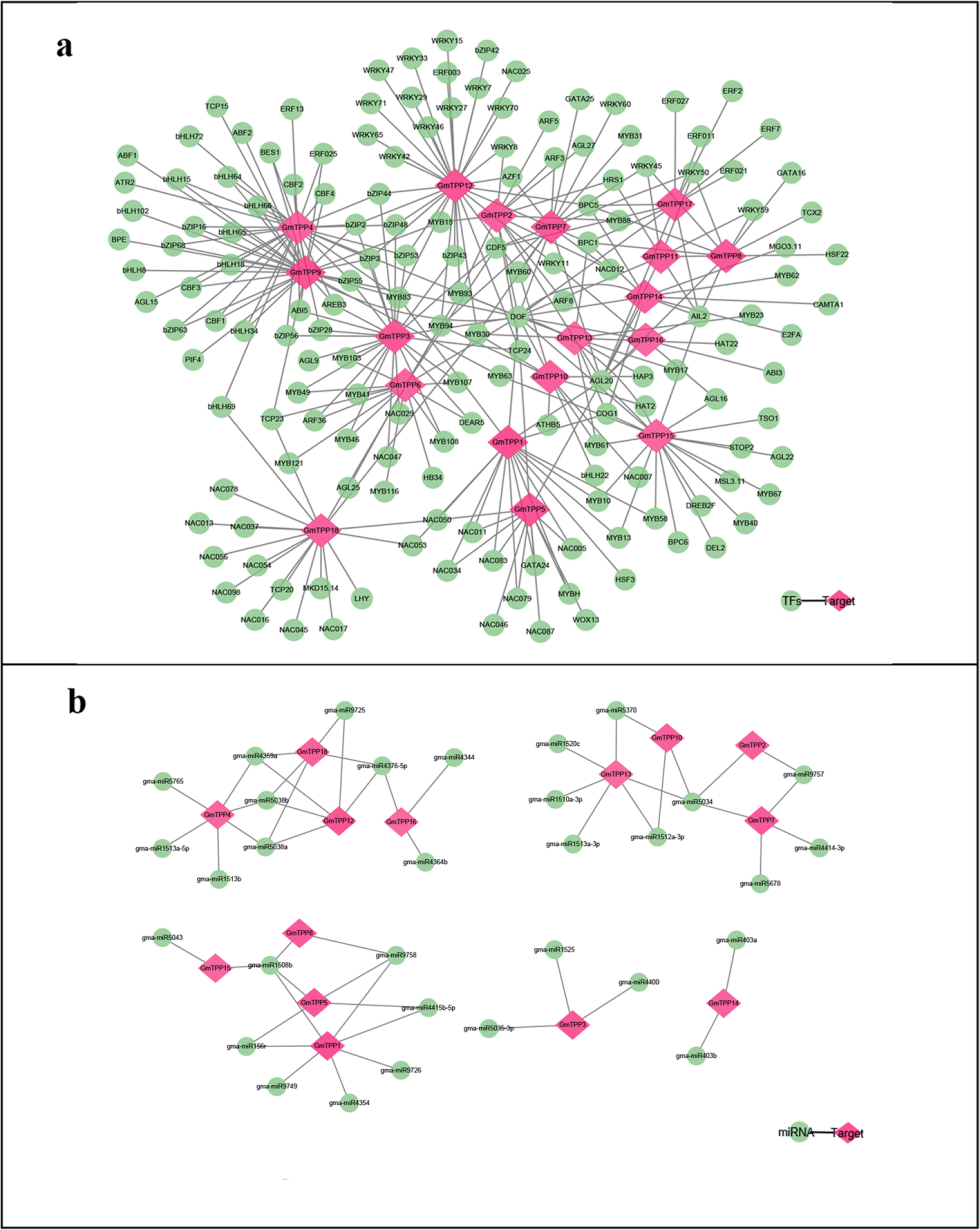
Prediction of transcription factors and miRNAs targeting *GmTPPs* genes. The green circles reflect the predicted TFs or miRNAs, and the pink circles depict the targeted TPP genes. The lines between the circles represent their connections

Orthology-based prediction of the transcription factor- *GmTPP* genes interactions revealed that the *GmTPP* genes interacted with many transcription factors, including *NAC*, *MYB, bZIP*, *bHLH*, *ERF*, and *WRKY*. *GmTPP14* and *GmTPP15* homologous to *AtTPPD* interacted with *COG*, *Dof*, *AGL*, *BPC,* and *AIL* (Fig. 9a). *GmTPP15* also interacted with multiple *MYBs* and one *WRKY*, while *GmTPP14* also interacted with *CAMTA*. In addition, *GmTPP5* interacted with multiple *NACs* and one *WOX* and *GmTPP2* with *TCP* and *ARF*. Some transcription factors also interacted with multiple genes. For example, *MYB* and *AGL* interacted with 13 *GmTPPs*, *NAC* with eight *GmTPPss*, and *Dof* with 14 *GmTPPs*.

The miRNAs are non-coding single-stranded RNA molecules with approximately 22 nucleotides in length. Encoded by endogenous genes, miRNAs regulate gene expression and responses to biotic and abiotic stresses in plants. The prediction of the miRNA-targeting gene networks of TPP family genes in soybean (Fig. 9b) identified 55 interaction relationships, including 33 miRNAs and 14 TPP family genes. Among the genes, gma-miR1508b regulated the expression of four *GmTPP* genes, *GmTPP1*/ *5*/ *8* /*15*. In addition, *GmTPP2*, *GmTPP7*, *GmTPP10,* and *GmTPP13* were targeted by the same miRNA (gma-miR5034). Seven miRNAs targeted *GmTPP1*, while *GmTPP4* and *GmTPP13* were each targeted by six miRNAs. Moreover, GMA-miR4379a, GMA-miR5038a, and gma-miR5038b regulated the expression of three *TPP* genes, *GmTPP4*/*12*/*18*, while gma-miR9758 and gma-miR1508b regulated the expression of three other TPP genes, *GmTPP5* /7 / *8*.

## Discussion

Trehalose plays a crucial role in plant growth, development, and response to abiotic stresses [24]. In plants, trehalose is synthesized through the TPS-TPP metabolic pathway, with T6P acting as an intermediate [25]. The *TPP* gene is a key regulator of trehalose synthesis in plants, and its transcription and expression are essential for mitigating plant damage caused by abiotic stress [26]. With the development of whole-genome sequencing, *TPP* gene have been identified in monocots and dicots, including 10 in *Arabidopsis* [11], 11 in maize [14], 13 in rice [12], 30 in wheat [27], 8 in tomato [28], and 79 in cotton [29]. The number of *GmTPP* genes among the different plant species, indicating that the *TPP* gene family is not conserved. In this study, we identified 18 potential TPP genes in the soybean genome, distributed across its 13 soybean chromosomes. Phylogenetic analysis classified these genes into three subgroups, which showed similar motifs, gene structures, and CREs. Additionally, the 18 *GmTPPs* exhibitedtypical characteristics of the Trehalose_PPase domain.

Exon–intron diversity plays an important role in the evolution of gene families [30]. Therefore, gene function can be elucidated by analyzing the gene structure [18]. Generally, functionally similar genes have similar exon–intron arrangements and protein structures [27]. In this study, the structure and number of exons and introns of *GmTPP* genes in the same subpopulation were similar, suggesting that genes in the same subpopulation might have similar functions. However, some differences were observed among subfamilies, possibly due to the evolutionary diversification of gene functions.

Gene duplication is one of the major drivers of the genome and genetic system evolution. It plays an essential role in evolution by facilitating the generation of new genes and gene functions [31]. There are four main evolutionary gene duplication events: whole genome duplication, tandem duplication, SD, and retro-transposition. Tandem and segmental duplications are the major gene replication events driving the plant gene family expansion [32]. Gene duplication events can leads to the emergence of novel functions [33]. In our analysis, we identified 14 pairs of fragment duplicate GmTPP genes in the soybean genome, indicating the importance of this duplication mechanism in expanding the TPP gene family. The *Ka/Ks* analysis further revealed that all the 14 duplicated *GmTPP* gene pairs had *Ka/Ks* ratios < 1, implying that they had undergone extensive purifying selection. Similar results have been observed in wheat and cotton [13]. Furthermore, the analysis collinearity patterns showed a closer relationship between soybean and wheat compared to other plant species, such as Arabidopsis, maize, tomato, and rice, suggesting that soybean and wheat are closely related. [34].

Homologous genes with higher collinearity usually have similar functions [35]. Therefore, the soybean TPPs that show collinearity with members of the *Arabidopsis* TPP family possibly have similar functions. Our results revealed that *GmTPP5*, *GmTPP14*, and *GmTPP15* were collinear with *AtTPPD*, suggesting that they may have similar functions. Previous studies revealed that the overexpression of *AtTPPD* improves plant tolerance to salt stress, increases plant starch levels under high soil salinity conditions, and regulates carbohydrate metabolism [15]. Therefore, *GmTPP5*, *GmTPP14*, and *GmTPP15* could have been involved in soybean response to salt stress and improving the salt resistance capacity of soybean. Furthermore, *GmTPP10* was collinear with *AtTPPE*, which is know to regulateROS accumulation and promote ABA-inhibited root elongation [36].

Furthermore, extensive studies have revealed that *TPP* genes are expressed in various tissue. For example, *TaTPPA*、*TaTPPB,* and *TaTPPG* are predominantly expressed in leaves, while *TaTPP9-6A/D* and *TaTPP8-5A/B/D* are highly expressed in the roots [27]. Similarly, the GmTPP gene family exhibits tissue-specific expression pattern. The expression of several *GmTPPs (GmTPP4/9/14/15)* show higher expression levels in root tissues, which is consistent with the expression patterns of their *Arabidopsis* homologs *AtTPPD*, *AtTPPG,* and *AtTPPI*, suggesting their involvement in the root development [37]. Furthermore, *GmTPP15* shows high expressed in flowers, indicating its potential rolein soybean flowering. The high expression of *GmTPP5*, *GmTPP7*, *GmTPP9*, *GmTPP10*, *and GmTPP13* in the leaves also suggested that they might play important roles in soybean leaf development. In soybean, *GmTPP13* was highly expressed in the pods at various developmental stages, implying it could be involved in pod development. However, *GmTPP14 and GmTPP15* were highly expressed in different tissues, indicating that they were involved in plant growth and development.

The predicted interaction network of GmTPP proteins with other transcription factors was also predicted based on an orthology-based method. The soybean TPP protein interacted with several transcription factors, including *NAC*, *MYB*, *WRKY*, and *bHLH*. These transcription factors are essential in regulating plant growth and development and stress responses. For example, *GmTPP2* interacted with *MYB93*, *MYB15*, and *WRKY8*. *AtMYB93* is a negative modulator of lateral root development in *Arabidopsis*. The *Atmyb93* mutant has a faster lateral root development and enhanced lateral root density [38]. *MYB15* positively regulates the cold tolerance of tomatoes through the CRT/DRE binding factors (CBF) pathway [39].

Furthermore, the expression of *WRKY8* induced by salt stress enhanced the plant tolerance to salt stress [40]. In rice, *MYB61*, which interacts with *GmTPP15,* improves grain yield and cold tolerance [41]. *GmTPP15* also interacts with *AGL16*, a negative regulator of stress response in *Arabidopsis. AGL16* mutations promote seed germination, root elongation, and increased plant resistance to salt stress [42].The prediction of miRNA-targeted *GmTPPs* provides insights into the complex regulatory network governing the expression of these genes. The identification of 33 miRNAs targeting 14 *GmTPPs* highlights the intricate regulation of 33 miRNAs through miRNA-mediated pathways, of which six genes were targeted by at least four miRNAs, revealing the complex regulation network of *GmTPPs*. This miRNA-GmTPP relationship provides insights into the precise genetic engineering of *GmTPPs* through miRNA mediation. Integrating the protein interaction network and miRNA-mediated pathways of *GmTPPs* could enhance our understanding of the regulation of stress responses and the growth and development of gene networks in soybean.

Under stress conditions, transcription factors are activated to bind specific cis-acting elements, regulating the expression of downstream stress-responsive genes. In this study, it was found that 89% of the *TPP* genes contained elements associated with anaerobic induction, which is relevant to waterlogging stress in soybean. However, regulating the *TPP* family genes may improve the resistance of soybean roots to waterlogging stress [43]. *GmTPP2*, *GmTPP10,* and *GmTPP17* genes contain defense and stress, drought-inducible, and low temperature-responsive elements, which may play a certain role in improving the drought and cold resistance of plants. In addition, the flavonoid biosynthetic regulation elements regulate the biosynthesis of flavonoids in plants. Flavonoid accumulation in plants is an important feature of plant resistance against environmental stresses. Besides, flavonoid compounds improve the plant resistance to peroxidation and ultraviolet radiation [44, 45].Furthermore, the presence of flavonoid biosynthetic regulatory elements in GmTPP1 and GmTPP15 suggests their involvement in flavonoid biosynthesis, which is important for plant resistance against environmental stresses. Moreover, more than half of the *TPP* genes contained ABA-responsive elements. In *Arabidopsis*, ABF2, a transcription factor responsive to ABA, directly binds the *AtTPPE* promoter, triggering its expression through ROS generation, which promotes root elongation and stomatal movement [36].

Previous studies revealed that trehalose production regulated by *TPP* genes alleviates the adverse effects of salt stress on plants [46, 47]. Herein, analysis of the response of the *GmTPP* genes to low-temperature, drought, saline-alkali, and exogenous trehalose stresses revealed that the gene expression profiles were similar after low-temperature and drought treatment. However, the expression induction, inhibition, or upregulation intensities were different. In this study, it was observed that *GmTPP2* and *GmTPP5* were significantly up-regulated under saline-alkali stress and trehalose immersion, suggesting their potential roles in improving salt and drought tolerance. *GmTPP5* had a collinear relationship with *AtTPPD*, which regulates plant tolerance to salt stress [15]. At the same time, *GmTPP10* was significantly up-regulated under drought stress. Collinearity analysis revealed that *GmTPP10* was collinear with *AtTPPE*, whose overexpression enhances the drought resistance of *Arabidopsis* [36].

Moreover, *GmTPP15* was significantly up-regulated under four abiotic stresses, indicating that its plays an important role in soybean response to abiotic stresses. Interestingly, the expression of most genes was up-regulated under the saline-alkaline treatment and exacerbated after trehalose immersion. For example, the expression ratio of *GmTPP2* was 2.2 under saline-alkaline stress for 72 h but increased to 22.9 after trehalose immersion. Similarly, the expression ratio of *GmTPP15* was increased from 15.2 to 20.5. These results are consistent to those found in tomatoes, where exogenous trehalose enhanced the expression of *SlTPPJ* and *SlTPPH* [48].

**Fig. 10:**
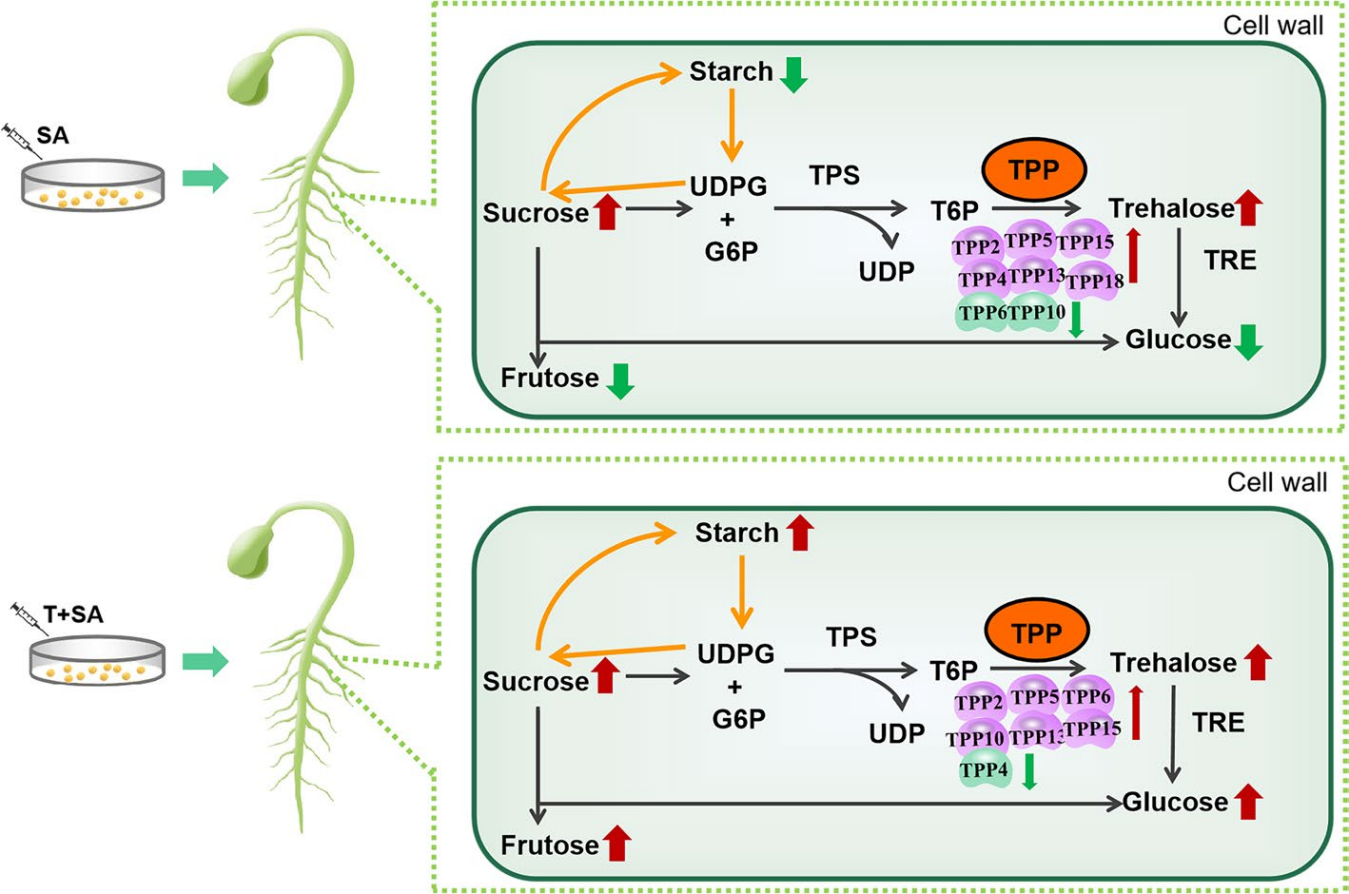
Trehalose metabolism in a plant cell, its role in carbohydrate metabolism and plant growth and development. Trehalose-6-phosphate (T6P) originates from the sucrose metabolic pathway. T6P is produced by the enzyme TREHALOSE-6-PHOSPHATE SYNTHASE (TPS) using UDP-glucose (UDPG) and glucose-6-phosphate (G6P), which is then transformed to trehalose by TREHALOSE-6-PHOSPHATE PHOSPHATASE (TPP) in the cytoplasm. Trehalose is cleaved into two molecules of glucose by TREHALASE1 (TRE1). The carbohydrate accumulation of plants was different under saline-alkali stress and exogenous trehalose

The *GmTPPs* may regulate soybean saline-alkali stress response by regulating trehalose metabolism. Trehalose plays a significant role in plant growth, development, and stress tolerance, especially tolerance to salt and drought stress [49], which explains the high trehalose concentrations in desert plants [50]. In this study, we drew a summary diagram of soybean response changes after salt-alkali stress and exogenous trehalose immersion (Fig. 10). Surprisingly, in this study, it was observed that sucrose and trehalose levels increased under saline-alkaline treatment alone, regardless of the altered expression of *GmTPP* genes and the decrease in glucose, fructose, and starch contents. Notably, high levels of soluble carbohydrate can positively regulated genes involved in sugar sensing and carbon metabolism under saline conditions [51]. For example, enhancing sucrose synthesis and reducing sucrose decomposition to increase sucrose content alleviated salt stress in salt-tolerant sweet sorghum [52]. In soybean sprouts, this phenomenon could be attributed to the reduced hydrolysis rate of sucrose and the conversion of trehalose to glucose under saline-alkaline stress. Trehalose immersion promoted the accumulation of carbohydrates, including endogenous trehalose, in soybean sprouts during saline-alkali stress. These results are consistent with previous reports [48, 53]. Additionally, analysis of ROS analysis revealed that saline-alkali sensitive varieties had higher ROS levels under saline-alkali stress, while trehalose-immersed seeds exhibited reduced ROS accumulation. These results demonstrates that trehalose immersion induces the expression of *GmTPP* gene, enhances the accumulation of carbohydrates in plants, and reduces the accumulation of ROS in plants, thereby improving the saline-alkali tolerance of soybeans.

The analysis of *TPP* family genes in soybean is essential for further studies on the response of soybean to environmental stress, soybean growth and development, and soybean yield improvement under stress conditions. Since plant stress resistance is an extremely complex physiological process controlled by multiple genes, the effect of transforming a single gene to improve plant stress resistance is significantly limited. However, adding and applying *TPP* gene promoters could resolve the shape changes of *TPP* gene plants [54]. Besides, using trehalose defense response to breed high-performing varieties under drought and salt stress is one of the research hotspots. With the deepening of our understanding of the specific biological function of trehalose, trehalose is expected to play a more important role in breeding resistance in crops.

## Conclusions

A total of 18 *GmTPP* genes were identified in the soybean genome and classified into three groups. The *GmTPPs* are highly conserved in soybean and involved in response to abiotic stresses. The results of RNA-seq showed that *GmTPP* genes are enriched in the trehalose biosynthesis pathway. Furthermore, exogenous trehalose application up-regulates *GmTPPs*, reducing ROS accumulation in plants and increasing the carbohydrate concentration under saline-alkaline stress, alleviating plant damage caused by saline-alkaline stress. The transcription factors-*GmTPPs* and miRNA-*GmTPPs* regulation relationships reveal the regulatory network of genes regulating the response to abiotic stress. The findings in this study unravel the biological activities of GmTPP proteins in developmental processes and stress responses in soybean, essential in breeding cultivars resistant to abiotic stresses.

## Methods

### Identification and classification of TPP genes in soybean

To identify the *TPP* family members in soybean, we downloaded the soybean reference genome assembly (*Glycine max Wm82.a2.v1*) and the gene annotation file from the Ensembl plants (http://jul2018-plants.ensembl.org/index.html). At the same time, the HMMER program was used to search the *TPP* family gene sequences against the Pfam (http://pfam.xfam.org/) library to identify the *TPP* genes in soybean. Subsequently, the TPP (Trehalose_PPase, PF02358) protein domains were identified using the SMART (http://smart.embl-heidelberg.de) and CDD (https://www.ncbi.nlm. nih.gov/Structure/cdd/wrpsb.Cgi) websites, and proteins lacking the TPP domain were removed [55]. Next, the isoelectric point and molecular weight of soybean TPP proteins were calculated using the bioinformatics resource portal, ExPASy (http://prosite.expasy.org/). The subcellular localization of GmTPP proteins was predicted using WoLF PSORT (https://wolfpsort.hgc.jp/). The *GmTPP* genes were named based on their location on the chromosomes after their physical locations were mapped using MapChart software (https://www.wur.nl/enshow/Mapchart/) [56]. Subsequently, the TPP protein sequences encoded by *TPP* genes in *Arabidopsis*, maize, wheat (*Triticum aestivum*), tomato (*Solanum lycopersicum*), and rice were downloaded from Phytozome13 or TAIR (https://www.arabidopsis.org/). All the TPP protein sequences in soybean obtained via the HMMER search were imported into MEGA X, and aligned. The ClustalW program in MEGA-X software was used to conduct multiple sequence alignment of TPP genes from multiple species, and a phylogenetic tree was constructed using a maximum likelihood (ML) with 1000 bootstraps. Next, a phylogenetic tree was generated in the bootstrap analysis using MEGA11. The phylogenetic tree was visualized and optimized using iTOL (https://itol.embl.de/) [57]. At the same time, the MEME online tool (http://meme.nbcr.net/meme/) was used to detect the GmTPPs motif, with 10 as the maximum number of lookups. Thereafter, the distribution map of the motifs was constructed by TBtools. The number of motifs was then compared among the GmTPPs to identify the group-conserved or group-specific signatures. Exon–intron structures of *TPP* genes in soybean were identified by the coding and the genomic sequences in *Glycine max* Wm82.a2.v1. The exon–intron structures were analyzed using Gene Structure Display Server (GSDS) (http://gsds.cbi.pku.edu.cn/). Finally, the expression data of GmTPPs in the specific tissues were obtained from the Phytozome database (https://phytozomnext.jgi.doe.gov/pz/). We predict the 3D structure of GmTPP protein by homology modeling method. First, we use the location-specific iterative BLAST algorithm (PSI-BLAST) to find the most similar homology in the PDB database (http://www.rcsb.org/), and then use the Swiss-Model interactive tool (https://swissmodel.expasy.org/interactive/) to predict the 3D structure of the GmTPP protein. In addition, in the save server (http://nihserver.mbi.ucla.edu/SAVES/) using PROCHECK test to detect the TPP protein 3D structure, and through Pymol software to display 3D structure.

### Homology analysis of the GmTPP family members

The soybean whole genome protein sequences were aligned using BLAST. Next, the whole genome collinearity analysis was performed using the MCScanX software. The non-synonymous (*Ka*) and synonymous (*Ks*) were used to assess the selection history and divergence time [58]. *Ka* and *Ks* substitution rates per *TPP* gene pair were calculated using the Simple *Ka/Ks* Calculator in TBtools. At the same time, MCScanX was used to analyze the gene duplication events of *GmTPPs,* which were subsequently visualized using TBtools.

### Analysis of the GmTPP promoters

The upstream 2000 bp coding DNA sequences (CDS) of *GmTPP*s were retrieved from the soybean genome and mapped against the PlantCare database (http://bioinformatics.psb.ugent.be/webtools/plantcare/) to identify the putative cis-regulatory elements in the promoter regions [59]. Subsequently, the putative cis-regulatory elements were visualized using Tbtool [60]. In addition, the *GmTPPs* expression levels in the specific tissues were obtained from the Phytozome database. Finally, TBtools and circos (version 0.69) (http://circos.ca/) were used to visualize the results [61].

### Plant materials and growth conditions

Inbred “Niuyanjing” (saline-alkali tolerant genotype, N) soybean seeds were provided by the National Coarse Cereals Engineering Research Center (Daqing, Heilongjiang, China). The seeds were surface-sterilized in 5% (v/v) sodium hypochlorite (NaClO) for 5 min and then thoroughly rinsed with distilled water. After sterilization, the seeds were assigned to five groups. Seeds in the first group were germinated in Petri dishes lined with a wet filter paper for 2 d in the dark at 25 °C before they were incubated in the refrigerator at 4 ℃ for 3 d. For seeds in groups two and three, drought and saline-alkali stresses were induced by soaking the seeds in 5% PEG-6000 (PEG treatment) or 10 mL mixed saline-alkali solution (NaCl, Na_2_CO_3_, NaHCO_3_, Na_2_SO_4_; molar ratio—1:1:9:9) of 160 mmol/L (saline-alkali treatment) for 3 d. In the fourth groups, the seeds were soaked in 10 mmol/L trehalose for 12 h, incubated in distilled water for 36 h, and finally treated with 160 mmol/L mixed saline-alkali solution for 3 d (T + SA treatment). The last group served as a control, kept the seeds in water. After 2 d of distilled water culture, hypocotyl, radicle and cotyledon were collected for tissue-specific expression analysis. The root tissue of soybean treated with 4 ℃, PEG, saline-alkali, and T + SA for 2 d were used for gene expression analysis, using those in water culture as the control. Each treatment had three biological replicates.

### RNA isolation and transcriptome and qRT-PCR analysis

The total RNA in the soybean tissues except the cotyledon was exacted 0, 12, 24, 48, and 72 h after being treated with 80 and 160 mmol/L saline-alkali solution using the TRIzol reagent (Invitrogen, Carlsbad, CA, United States) following the manufacturer’s instructions. The quantity of the extracted RNA was detected using NanoDrop (Thermo, Carlsbad, CA, United States). The OD_260/280_ of the extracted RNA was between 1.8 and 2.2, while 28S/ 18S was greater than 1, indicating good quality. The RNA integrity was assessed using an Agilent Bioanalyzer 2100 (Agilent Technologies, Carlsbad, California). The qualified RNA was used for RNA library construction. At the same time, we used these samples for transcriptome sequencing and identification of differentially expressed gene RNA-seq data validation.

The RNA sequencing was performed on the Illumina HiSeq platform at BMK (Biomarker Technologies, Beijing, China) with three biological replicates per treatment. The raw sequencing data were filtered to remove adapter sequences and obtain high-quality clean reads. Reads with > 50% of the bases having a Q-value ≤ 5 were retained. The clean sequences of each library were mapped to the reference sequence (https://phytozomnext.jgi.doe.gov/info/Gmax_Wm82_a2_v1) using TopHat (http://tophat.cbcb.umd.edu/). In addition, the differentially expressed genes (DEGs) were determined using default parameters, including a false discovery rate (FDR) < 0.05 and a logarithm two-fold change |log2FC|≥ 2. Subsequently, the Gene Ontology (GO) and Kyoto Encyclopedia of Genes and Genomes (KEGG) enrichment analysis and annotation of the DEGs were performed using the KEGG pathway database (http://www.genome.jp/keg).

Moreover, the total RNA from the T + SA treatment was reverse transcribed using oligo (dT) primer and SuperScript Reverse Transcriptase (TaKaRa, Nanjing, China). qRT-PCR was then performed using SYBR green (TaKaRa Biotechnology) on Roche Cycler 480II system (Roche, Roche Diagnostics, Switzerland). The gene-specific primers (Additional file 7. Table S5) for each *GmTPPs* were designed using Premier 5.0 software [62]. *GmACTIN-11* was used as the internal control gene. Each treatment had three biological and three independent replicates. Finally, the relative gene expression of *GmTPPs* was analyzed using the 2 ^−ΔΔc^(t) method [63].

### Nitro blue tetrazolium (NBT), diaminobenzidine (DAB) and trypan blue (TB) staining

The soybean sprouts were treated with different stress treatments for 3 d and stained with NBT, DAB and TB dye solutions according to previously reported methods [64]. NBT, DAB and TB stainings were repeated thrice.

### Carbohydrate and trehalose contents analysis

Frozen tissues (100–150 mg) were weighed and ground for 30 to 60 s. Sugars (Sucrose, fructose, and glucose) were then extracted following the method described by Lunn et al. (2006) with minor modifications [46]. At the same time, starch was extracted from the precipitate during the extraction of soluble sugars and quantified through Glc analysis during hydrolysis. To generate the standard curve, standard glucose, fructose, and sucrose (Sigma, purity ≥ 99.9%) samples were accurately weighed, and each prepared into a mother liquor (10 mg/mL). The standard mixture was then diluted into a standard solution. Finally, the standard curve of the different sugars with the abscissa and ordinate as the mass concentration and chromatographic peak area, respectively, was generated [48]. The trehalose content was measured using a trehalose kit (Solarbio, Beijing, China).

### Prediction of the transcription factors and miRNAs

The transcription factors interacting with *GmTPP* genes were predicted using the Plant Transcriptional Regulatory Map website (http://plantregmap.gao-lab.org/network.php) based on the homologous genes between soybean and *Arabidopsis*. At the same time, the psRNATarget server (https://www.zhaolab.org/psRNATarget/) was used to predict the target relationships between miRNA and *TPP* genes in soybean [65]. Briefly, the CDS sequence of *TPP* genes in soybean was submitted as target candidates against the published miRBase of soybean. The default option was selected for other parameters. The results were visualized using Cytoscape [66]

### Statistical analyses

Each experiment was performed in triplicates. All data were analyzed and presented as mean ± standard deviation (SD). The data were analyzed by student's t-test at *P* < 0.05 (statistically significant and *P* < 0.01 (highly statistically significant) levels of significance.

### Supplementary Information

**Additional file1: Figure S1.** Calculated ramachandran plots for modeled 3D structures of GmTPP.

**Additional file 2: Table S1.** TPP protein sequences used by this study.

**Additional file 3**:** Table S2.** All protein sequences used by this study.

**Additional file 4: Table S3.** KaKs data of TPP genes of soybean, Arabidopsis, maize, wheat, rice and tomato.

**Additional file 5: Table S4.** Organization of cis-acting regulatory elements in soybean GmTPP gene family promoters.

**Additional file 6: Figure S2.** Results of qRT-PCR in different tissue parts of GmTPP gene.

**Additional file 7: Table S5.** Primers used in this research.

## Data Availability

All data generated or analyzed during this study are included in this article and its supplementary information files. All databases used in this study are open for public and the links are as follows: Ensembl plants: http://jul2018-plants.ensembl.org/index.html Pfam: http://pfam.xfam.org CDD: https://www.ncbi.nlm .nih.gov/Structure/cdd/wrpsb.Cgi. ExPASy: http://prosite.expasy.org/ WoLF PSORT: https://wolfpsort.hgc.jp MapChart: https://www.wur.nl/enshow/Mapchart/ TAIR: https://www.arabidopsis.org/ iTOL: https://itol.embl.de/ MEME: http://meme.nbcr.net/meme/ GSDS: http://gsds.cbi.pku.edu.cn/ Phytozome 13: https://phytozomnext.jgi.doe.gov/pz/. PlantCare: https://bioinformatics.psb.ugent.be/webtools/plantcare/html/ TopHat: http://tophat.cbcb.um d.edu/ KEGG database: http://www.genome.jp/kegg psRNATarget server: https://www.zhaolab.org/psRNATarget/

## References

[1] Bansal R, Mian MAR, Mittapalli O, Michel AP (2013). Molecular characterization and expression analysis of soluble trehalase gene in Aphis glycines, a migratory pest of soybean. Bull Entomol Res.

[2] Tang B, Wang S, Wang S-G, Wang H-J, Zhang J-Y, Cui S-Y (2018). Invertebrate trehalose-6-phosphate synthase gene: genetic architecture, biochemistry, physiological function, and potential applications. Front Physiol.

[3] Fichtner F, Lunn JE (2021). The role of trehalose 6-phosphate (Tre6P) in plant metabolism and development. Annu Rev Plant Biol.

[4] Kataya ARA, Elshobaky A, Heidari B, Dugassa N-F, Thelen JJ, Lillo C (2020). Multi-targeted trehalose-6-phosphate phosphatase I harbors a novel peroxisomal targeting signal 1 and is essential for flowering and development. Planta.

[5] Saddhe AA, Manuka R, Penna S (2021). Plant sugars: Homeostasis and transport under abiotic stress in plants. Physiol Plant.

[6] Deyanira Q-M, Estrada-Luna AA, Altamirano-Hernández J, PeñaCabriales JJ, de Oca-Luna RM, Cabrera-Ponce JL (2012). Use of trehalose metabolism as a biochemical marker in rice breeding. Mol Breeding.

[7] Espasandin FD, Calzadilla PI, Maiale SJ, Ruiz OA, Sansberro PA (2018). Overexpression of the Arginine Decarboxylase gene improves tolerance to salt stress in Lotus tenuis plants. J Plant Growth Regul.

[8] Cabib LFL (1958). The biosynthesis of trehalose phosphate. J Biol Chem.

[9] Svanström Å, Leeuwen MRV, Dijksterhuis J, Melin P (2014). Trehalose synthesis in Aspergillus Niger: characterization of six homologous genes, all with conserved orthologs in related species. BMC Microbiol.

[10] Satoh-Nagasawa N, Nagasawa N, Malcomber S, Sakai H, Jackson D (2006). A trehalose metabolic enzyme controls inflorescence architecture in maize. Nature.

[11] Delorge I, Figueroa CM, Feil R, Lunn JE, Dijck PV (2015). Trehalose-6-phosphate synthase 1 is not the only active TPS in Arabidopsis thaliana. Biochem J.

[12] Jin Q, Hu X, Li X, Wang B, Wang Y, Jiang H, Mattson N, Xu Y (2016). Genome-wide identification and evolution analysis of Trehalose-6-phosphate synthase gene family in Nelumbo nucifera. Front Plant Sci.

[13] Wang W, Cui H, Xiao X, Wu B, Sun J, Zhang Y, Yang Q, Zhao Y, Liu G, Qin T (2022). Genome-wide identification of cotton (Gossypium spp.) Trehalose-6-Phosphate Phosphatase (TPP) gene family members and the role of GhTPP22 in the response to drought stress. Plants (Basel).

[14] Acosta-Pérez P, Camacho-Zamora BD, Espinoza-Sánchez EA, Gutiérrez-Soto G, Zavala-García F, Abraham-Juárez MJ, Sinagawa-García SR (2020). Characterization of Trehalose-6-phosphate synthase and Trehalose-6-phosphate phosphatase genes and analysis of its differential expression in maize (Zea mays) seedlings under drought stress. Plants.

[15] Krasensky J, Broyart C, Rabanal FA, Jonak C (2014). The redox-sensitive chloroplast trehalose-6-phosphate phosphatase AtTPPD regulates salt stress tolerance. AntioxidRedox Sign.

[16] Carillo P, Feil R, Gibon Y, Satoh-Nagasawa N, Jackson D, Bläsing OE, Stitt M, Lunn JE (2013). A fluorometric assay for trehalose in the picomole range. Plant Methods.

[17] Lin Q, Yang J, Wang Q, Zhu H, Chen Z, Dao Y, Wang K (2019). Overexpression of the trehalose-6-phosphate phosphatase family gene AtTPPF improves the drought tolerance of Arabidopsis thaliana. BMC Plant Biol.

[18] Li Y, Chen D, Luo S, Zhu Y, Jia X, Duan Y, Zhou M (2019). Intron-mediated regulation of β-tubulin genes expression affects the sensitivity to carbendazim in Fusarium graminearum. Curr Genet.

[19] Ge L-F, Chao AY, Shi M, Zhu MZ, Gao JP, Lin HX (2008). Overexpression of the trehalose-6-phosphate phosphatase gene OsTPP1 confers stress tolerance in rice and results in the activation of stress responsive genes. Planta.

[20] Jiang D, Chen W, Gao J, Yang F, Zhuang C (2019). Overexpression of the trehalose-6-phosphate phosphatase OsTPP3 increases drought tolerance in rice. Plant Biotechnol Rep.

[21] Nuccio ML, Wu J, Mowers R, Zhou H-P, Meghji M, Primavesi LF, Paul MJ, Xi Chen YG, Haque E, Basu SS (2015). Expression of trehalose-6-phosphate phosphatase in maize ears improves yield in well-watered and drought conditions. Nat Biotechnol.

[22] Rastegar Z, Aghighi M, Kandi S (2011). The effect of salinity and seed size on seed reserve utilization and seedling growth of soybean. Int J Agron Plant Prod.

[23] Wang F, Chen H-W, Li Q-T, Wei W, Li W, Zhang W-K, Ma B, Bi Y-D, Lai Y-C, Liu X-L (2015). GmWRKY27 interacts with GmMYB174 to reduce expression of GmNAC29 for stress tolerance in soybean plants. Plant J.

[24] Delorge I, Janiak M, Carpentier S, Dijck PV (2014). Fine tuning of trehalose biosynthesis and hydrolysis as novel tools for the generation of abiotic stress tolerant plants. Front Plant Sci.

[25] Lin Y, Zhang J, Gao W, Chen Y, Li H, Lawlor DW, Paul MJ, Pan W (2017). Exogenous trehalose improves growth under limiting nitrogen through upregulation of nitrogen metabolism. BMC Plant Biol.

[26] Zhang P, He Z, Tian X, Gao F, Xu D, Liu J, Wen W, Fu L, Li G, Sui X, et al. Cloning of TaTPP-6AL1 associated with grain weight in bread wheat and development of functional marker. Mol Breed. 2017;37:78.

[27] Du L, Li S, Ding L, Cheng X, Kang Z, Mao H (2022). Genome-wide analysis of trehalose-6-phosphate phosphatases (TPP) gene family in wheat indicates their roles in plant development and stress response. BMC Plant Biol.

[28] Mollavali M, Börnke F (2022). Characterization of Trehalose-6-phosphate synthase and trehalose-6-Phosphate phosphatase genes of tomato (Solanum lycopersicum L.) and analysis of their differential expression in response to temperature. Int J Mol Sci.

[29] Wang W, Cui H, Xiao X, Wu B, Sun J, Zhang Y, Yang Q, Zhao Y, Liu G (2022). 1 TQ: Trehalose-6-Phosphate Phosphatase (TPP) gene family members and the role of GhTPP22 in the response to drought stress. Plants (Basel).

[30] Qi L, Chen L, Wang C, Zhang S, Yang Y, Liu J, Li D, Song J, Wang R (2020). Characterization of the auxin efflux transporter PIN proteins in pear. Plants.

[31] Wang Y, Li Y, Zhou F, Zhang L, Gong J, Cheng C, Chen J, Lou Q (2023). Genome-wide characterization, phylogenetic and expression analysis of Histone gene family in cucumber (Cucumis sativus L.). Int J Biol Macromol.

[32] Cannon SB, Mitra A, Baumgarten A, Young ND, May G (2004). The roles of segmental and tandem gene duplication in the evolution of large gene families in Arabidopsis thaliana. BMC Plant Biol.

[33] Li Z, Zhang C, Guo Y, Niu W, Wang Y, Xu Y (2017). Evolution and expression analysis reveal thepotential role of the HD-zip gene family in regulation of embryo abortion in grapes (Vitis vinifera L.). BMC Genomics.

[34] Heidari P, Abdullah, Faraji S, Poczai P. Magnesium transporter gene family: genomewide identification and characterization in theobroma cacao, corchorus capsularis, and gossypium hirsutum of family malvaceae. Agronomy. 2021;11:49.

[35] Moore RC, Purugganan MD (2003). The early stages of duplicate gene evolution. Proc Natl Acad Sci U S A.

[36] Wang W, Chen Q, Shouming XU, Wen-Cheng L, Zhu X, Song CP (2020). Trehalose-6-Phosphate phosphatase E modulates ABA-controlled root growth and stomatal movement in Arabidopsis. J Integr Plant Biol.

[37] Vandesteene L, López-Galvis L, Vanneste K, Feil R, Maere S, Lammens W, Rolland F, Lunn JE, Avonce N, Beeckman T (2012). Expansive evolution of the Trehalose-6-Phosphate Phosphatase gene family in Arabidopsis. Plant Physiol.

[38] Gibbs DJ, Voß U, Harding SA, Fannon J, Moody LA, Yamada E, Swarup K, Nibau C, Bassel GW, Choudhary A (2014). AtMYB93 is a novel negative regulator of lateral root development in Arabidopsis. New Phytol.

[39] Zhang L, Jiang X, Liu Q, Ahammed GJ, Lin R, Wang L, Shao S, Yu J, Zhou Y (2020). The HY5 and MYB15 transcription factors positively regulate cold tolerance in tomato via the CBF pathway. Plant Cell Environ.

[40] Hu Y, Chen L, Wang H, Zhang L, Wang F, Yu D (2013). Arabidopsis transcription factor WRKY8 functions antagonistically with its interacting partner VQ9 to modulate salinity stress tolerance. Plant J.

[41] Gao Y, Xu Z, Zhang L, Li S, Wang S, Yang H, Liu X, Zeng D, Liu Q, Qian Q (2020). MYB61 is regulated by GRF4 and promotes nitrogen utilization and biomass production in rice. Nat Commun.

[42] Zhao P-X, Zhang J, Chen S-Y, Wu J, Xia J-Q, Sun L-Q, Ma S-S, Xiang C-B (2021). Arabidopsis MADS-box factor AGL16 is a negative regulator of plant response to salt stress by downregulating salt-responsive genes. New Phytol.

[43] Vitor SC, Sodek L (2019). Products of anaerobic metabolism in waterlogged roots of soybean are exported in the xylem. Plant Sci.

[44] Beatriz L, Nascimento DS, Tattini M (2022). Beyond Photoprotection: the multifarious roles of flavonoids in plant terrestrialization. Int J Mol Sci.

[45] Sharma A, Shahzad B, Rehman A, Bhardwaj R, Landi M, Zheng B (2019). Response of Phenylpropanoid pathway and the role of polyphenols in plants under abiotic stress. Molecules.

[46] Lunn JE, Delorge I, Figueroa CM, Dijck PV, Stitt M (2014). Trehalose metabolism in plants. Plant J.

[47] Islam MO, Kato H, Shima S, Tezuka D, Matsui H, Imai R (2019). Functional identification of a rice trehalase gene involved in salt stress tolerance. Gene.

[48] Yang Y, Yao Y, Li J, Zhang J, Zhang X, Hu L, Ding D, Bakpa EP, Xie J (2022). Trehalose alleviated salt stress in tomato by regulating ROS metabolism, photosynthesis, Osmolyte synthesis, and Trehalose metabolic pathways. Front Plant Sci.

[49] Jang I-C, Oh S-J, Seo J-S, Choi W-B, Song SI, Kim CH, Kim YS, Seo H-S, Choi YD, Nahm BH (2003). Expression of a bifunctional fusion of the Escherichia coli genes for Trehalose-6-phosphate synthase and Trehalose-6-Phosphate Phosphatase in transgenic rice plants increases Trehalose accumulation and abiotic stress tolerance without stunting growth. Plant Physiol.

[50] Ambastha V, Tiwari BS (2015). Cellular water and anhydrobiosis in plants. J Plant Growth Regul.

[51] Wang X, Wang M, Huang Y, Zhu P, Qian G, Zhang Y, Liu Y, Zhou J, Li AL (2023). Genome-wide identification and analysis of stress response of Trehalose-6-Phosphate synthase and Trehalose-6-Phosphate Phosphatase genes in Quinoa. Int J Mol Sci.

[52] Sui N, Yang Z, Liu M, Wang B (2015). Identification and transcriptomic profiling of genes involved in increasing sugar content during salt stress in sweet sorghum leaves. BMC Genomics.

[53] Sm S (2019). Physiological role of trehalose on enhancing salinity tolerance of wheat plant. Bull Natl Res Cent.

[54] Karim S, Aronsson H, Ericson H, Pirhonen M, Leyman B, Welin B, Mäntylä E, Palva ET, Dijck PV, Holmström K-O (2007). Improved drought tolerance without undesired side effects in transgenic plants producing trehalose. Plant Mol Biol.

[55] Lu S, Wang J, Chitsaz F, Derbyshire MK, Geer RC, Gonzales NR, Gwadz M, Hurwitz DI, Marchler GH, Song JS (2020). CDD/SPARCLE: the conserved domain database in 2020. Nucleic Acids Res.

[56] Voorrips RE (2002). MapChart: software for the graphical presentation of linkage maps and QTLs. J Hered.

[57] Letunic I, Bork P (2021). Interactive Tree Of Life (iTOL) v5: an online tool for phylogenetic tree display and annotation. Nucleic Acids Res.

[58] Nei M, Gojobori T (1986). Simple methods for estimating the numbers of synonymous and nonsynonymous nucleotide substitutions. Mol Biol Evol.

[59] Lescot M, Déhais P, Thijs G, Marchal K, Moreau Y, Peer YVD, Rouzé P, Rombauts S (2002). PlantCARE, a database of plant cis-acting regulatory elements and a portal to tools for in silico analysis of promoter sequences. Nucleic Acids Res.

[60] Chen C, Chen H, Zhang Y, Thomas HR, Frank MH, He Y, Xia R (2020). TBtools: an integrative toolkit developed for interactive analyses of big biological data. Mol Plant.

[61] Rasche H, Hiltemann S (2020). Galactic circos: user-friendly circos plots within the galaxy platform. Gigascience.

[62] Singh VK, Mangalam AK, Dwivedi S, Naik S (1998). Primer premier: program for design of degenerate primers from a protein sequence. Biotechniques.

[63] Giulietti A, Overbergh L, Valckx D, Decallonne B, Bouillon R, Mathieu C (2001). An overview of real-time quantitative PCR: applications to quantify cytokine gene expression. Methods.

[64] Kumar D, Yusuf MA, Singh P, Sardar M, Sarin N: Histochemical detection of superoxide and H2O2 accumulation in Brassica juncea seedlings. Bio-Protocol 2014 4.

[65] Dai X, Zhuang Z, Zhao PX. psRNATarget: A plant small RNA target analysis server (2017 release). Nucleic Acids Res. 2018;46:45–54. 10.1093/nar/gky316 PMC6030838 29718424

[66] Otasek D, Morris JH, Bouças J, Pico AR, Demchak B (2019). Cytoscape Automation: Empowering workflow-based network analysis. Genome Biol.

