# Supplementary figures and images for: Genome- and transcriptome-wide identification of trehalose-6-phosphate phosphatases (TPP) gene family and their expression patterns under abiotic stress and exogenous trehalose in soybean
Source: BMC Plant Biol. 2023 Dec 12;23:641. doi: 10.1186/s12870-023-04652-7 (PMC10714469; doi:10.1186/s12870-023-04652-7)

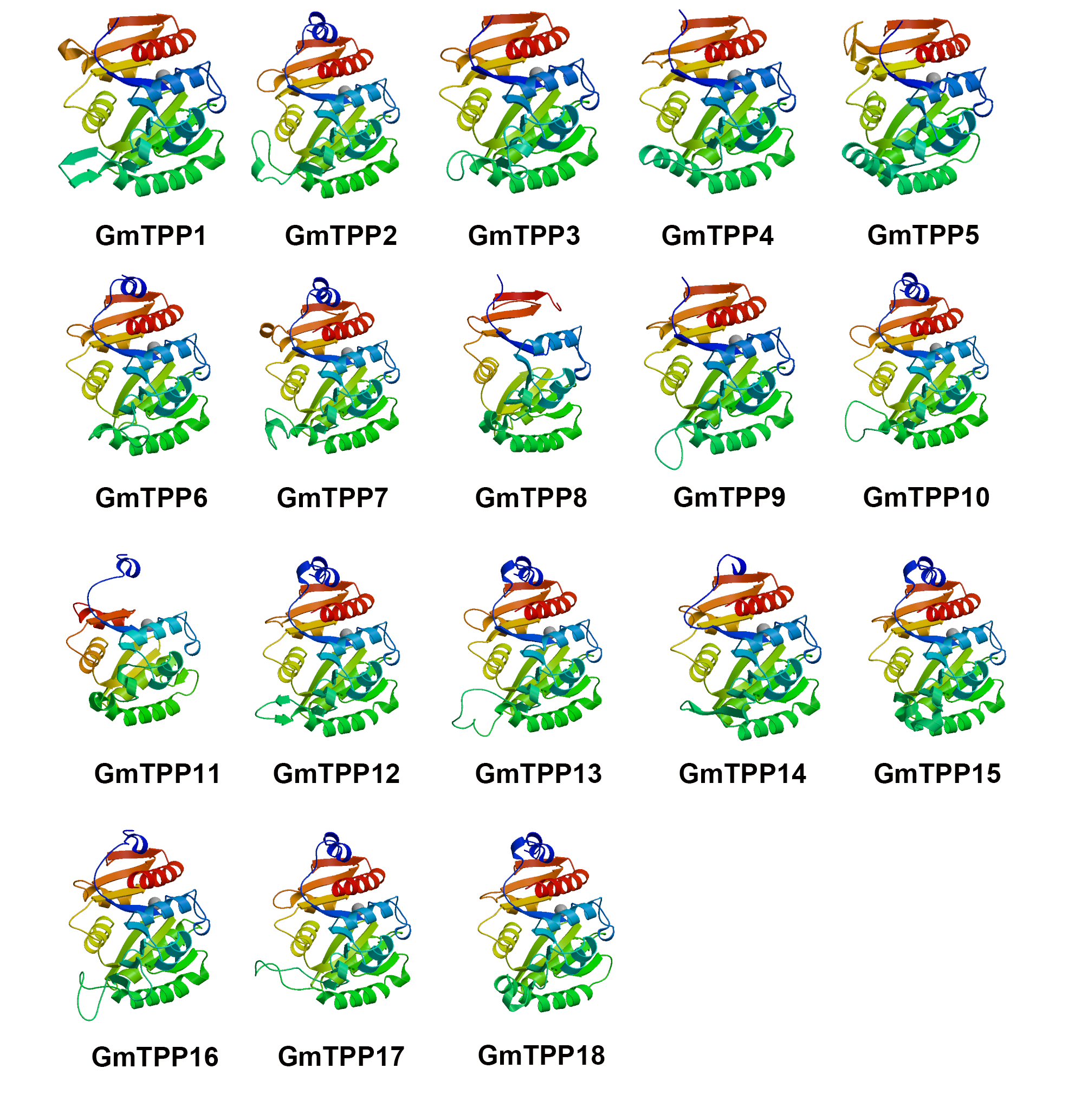

Supplement: Supplementary file 1 — Additional file1: Figure S1. Calculated ramachandran plots for modeled 3D structures of GmTPP. [file 12870_2023_4652_MOESM1_ESM.tif]

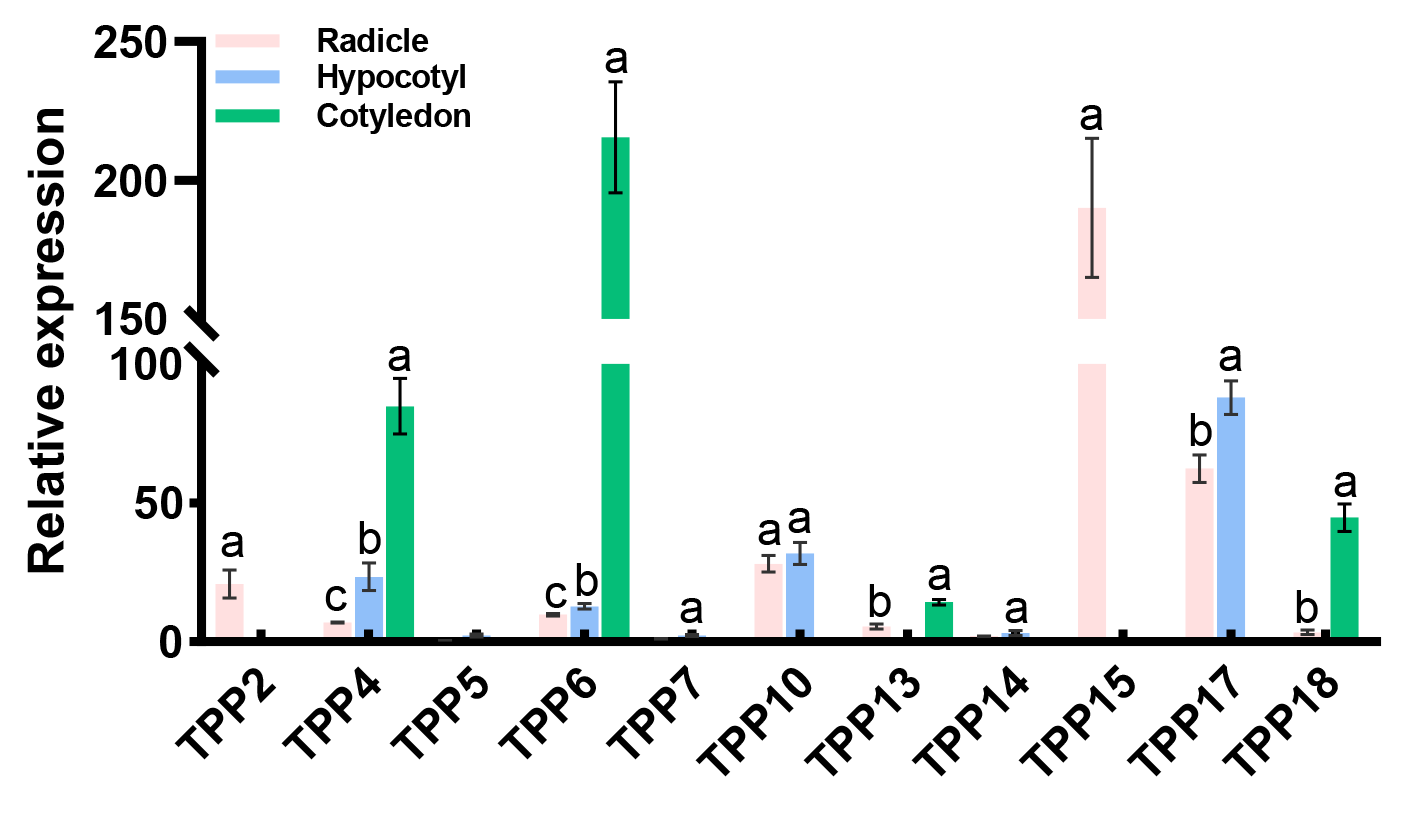

Supplement: Supplementary file 6 — Additional file 6: Figure S2. Results of qRT-PCR in different tissue parts of GmTPP gene. [file 12870_2023_4652_MOESM6_ESM.tif]
